# Clinicopathological impacts of DNA methylation alterations on pancreatic ductal adenocarcinoma: prediction of early recurrence based on genome-wide DNA methylation profiling

**DOI:** 10.1007/s00432-021-03541-6

**Published:** 2021-02-26

**Authors:** Yutaka Endo, Mao Fujimoto, Nanako Ito, Yoriko Takahashi, Minoru Kitago, Masahiro Gotoh, Nobuyoshi Hiraoka, Teruhiko Yoshida, Yuko Kitagawa, Yae Kanai, Eri Arai

**Affiliations:** 1 Department of Pathology, Keio University School of Medicine, 35 Shinanomachi, Shinjuku-ku, Tokyo, 160-8582 Japan; 2 Department of Surgery, Keio University School of Medicine, Tokyo, 160-8582 Japan; 3 Bioscience Department, Solution Knowledge Center, Mitsui Knowledge Industry Co., Ltd., Tokyo, 105-6215 Japan; 4 Fundamental Innovative Oncology Core Center, National Cancer Center Research Institute, Tokyo, 104-0045 Japan; 5 Department of Pathology and Clinical Laboratory, National Cancer Center Hospital, Tokyo, 104-0045 Japan

**Keywords:** DNA methylation, Infinium assay, Ductal adenocarcinoma, Pancreas, Early recurrence

## Abstract

**Purpose:**

The present study was conducted to clarify the clinicopathological impacts of DNA methylation alterations on pancreatic ductal adenocarcinoma (PDAC).

**Methods:**

Genome-wide DNA methylation screening was performed using the Infinium HumanMethylation450 BeadChip, and DNA methylation quantification was verified using pyrosequencing. We analyzed fresh-frozen tissues from an initial cohort (17 samples of normal control pancreatic tissue [C] from 17 patients without PDAC, and 34 samples of non-cancerous pancreatic tissue [N] and 82 samples of cancerous tissue [T] both obtained from 82 PDAC patients) and formalin-fixed paraffin-embedded T samples from 34 patients in a validation cohort.

**Results:**

The DNA methylation profiles of N samples tended to differ from those of C samples, and 91,907 probes showed significant differences in DNA methylation levels between C and T samples. Epigenetic clustering of T samples was significantly correlated with a larger tumor diameter and early recurrence (ER), defined as relapse within 6 months after surgery. Three marker CpG sites, applicable to formalin-fixed paraffin-embedded surgically resected materials regardless of their tumor cell content, were identified for prediction of ER. The sensitivity and specificity for detection of patients belonging to the ER group using a panel combining these three marker CpG sites, including a CpG site in the *CDK14* gene, were 81.8% and 71.7% and 88.9% and 70.4% in the initial and validation cohorts, respectively.

**Conclusion:**

These findings indicate that DNA methylation alterations may have a clinicopathological impact on PDAC. Application of our criteria will ultimately allow prediction of ER after surgery to improve the outcome of PDAC patients.

**Supplementary Information:**

The online version contains supplementary material available at 10.1007/s00432-021-03541-6.

## Introduction

Pancreatic ductal adenocarcinoma (PDAC) is a devastating disease that has become one of the major causes of cancer-related death in the United States (Siegel et al. 2016) and Japan (Egawa et al. 2012). Even in patients with resectable cancer diagnosed early, the cancer recurs after a short interval up to 80% of cases (Groot et al. 2018). Despite recent developments in pre- and postoperative management (Conroy et al. 2018; Motoi et al. 2019; Murphy et al. 2018; Neoptolemos et al. 2017; Sinn et al. 2017; Uesaka et al. 2016), there is still a significant need to develop more effective strategies, especially those based on molecular profiling, for stratification of patients with PDAC to improve their prognosis.

Early recurrence (ER) of loco-regional and/or distant metastases in PDAC after curative resection has been a serious therapeutic challenge. The median survival time for patients who develop distant metastases within 6 months after surgery is reportedly less than 12 months (Groot et al. 2019; Matsumoto et al. 2015). Therefore, patients in this group with more aggressive PDACs need to be identified before relapse occurs. Previous studies have suggested that tumor size, carbohydrate antigen 19-9 (CA19-9), histologic type, lymph-node metastasis, and microvascular or perineurial invasion are predictive factors for recurrence of PDAC after surgery (Groot et al. 2019; Kurahara et al. 2018; Matsumoto et al. 2015; Nishio et al. 2017; Sugiura et al. 2012). However, such factors alone are not yet sufficiently predictive of ER, and an extensive search for molecular-based biomarkers of ER risk in PDAC is warranted.

Aberrant DNA methylation is one of the most important epigenetic alterations occurring during carcinogenesis, resulting in chromosomal instability and altering the expression levels of tumor-related genes in different organs exposed to various carcinogenetic factors (Baylin et al. 2016; Jones et al. 2016; Kuramoto et al. 2017; Makabe et al. 2019; Ohara et al. 2017; Tsumura et al. 2019). With regard to pancreatic carcinogenesis, we have reported that cumulative DNA methylation of tumor-related genes, such as *BRCA1, APC, CDKN2A*, and *TIMP3*, is associated with overexpression of DNA methyltransferase (DNMT) 1 (Peng et al. 2005), the major DNMT, even in peripheral pancreatic duct epithelia with an inflammatory background (Peng et al. 2006), which may be at the precancerous stage in the context of chronic pancreatitis. The average number of methylated tumor-related genes increases further during progression from well to poorly differentiated ductal adenocarcinoma (Peng et al. 2005), suggesting that DNA methylation alterations continuously participate in multistage pancreatic carcinogenesis. In addition, based on genome-wide DNA methylation analysis using a bacterial artificial chromosome (BAC) array, we have confirmed the feasibility of DNA methylation diagnostics and prognostication of pancreatic cancers (Gotoh et al. 2011). However, such procedures are not easily applicable in a clinical situation because of the need for simultaneous quantification of DNA methylation status at numerous CpG sites on multiple BAC clones.

Recently, appropriate single-CpG-resolution genome-wide DNA methylation screening methods, e.g., the Infinium assay (Bibikova et al. 2009), have been introduced for analysis of various human tissue specimens. Although many previous studies using such screening methods, including those based on data from the International Cancer Genome Consortium (ICGC) (https://icgc.org) and The Cancer Genome Atlas (TCGA) (https://www.cancer.gov/about-nci/organization/ccg/research/structural-genomics/tcga), have revealed the DNA methylation profiles of PDACs (Kim et al. 2015; Liu et al. 2019; Nones et al. 2014; Zhang et al. 2019) and diagnostic criteria based on DNA methylation, especially those applicable to prognostication, are still far from established.

In the present study, to further clarify the clinicopathological impacts of DNA methylation profiles on PDAC, we performed single-CpG-resolution genome-wide DNA methylation screening using the Infinium assay in an initial cohort of 133 fresh-frozen pancreatic tissue samples and a meticulous follow-up survey of outcome. The reliability of our prognostication criteria was then investigated in a validation cohort comprising 36 additional samples of microdissected formalin-fixed paraffin-embedded tissue samples.

## Materials and methods

### Patients and tissue samples

The initial cohort consisted of 34 samples of non-cancerous pancreatic tissue (N) and 82 samples of the corresponding cancerous tissue (T) obtained from specimens that had been surgically resected during pancreatectomy from 82 patients with PDAC at the National Cancer Center Hospital, Tokyo, Japan. For comparison, 17 samples of normal control pancreatic tissue (C) obtained from 17 patients without PDAC who underwent pancreatectomy for metastasis of renal cell carcinoma (1 patient), adenocarcinoma of the papilla of Vater (6 patients), gallbladder (3 patients) or bile duct (1 patient), abscess (1 patient), serous cystadenoma (1 patient), mucinous cystadenoma (1 patient), solid-pseudopapillary neoplasm (1 patient) and endocrine tumor (1 patient) of the pancreas, and lymphoplasmacytic pancreatitis (1 patient) were examined. The validation cohort consisted of 36 T samples obtained from specimens that had been surgically resected by pancreatectomy from 36 patients with PDAC at Keio University Hospital. This study was approved by the Ethics Committees of the National Cancer Center and Keio University, and was performed in accordance with the Declaration of Helsinki. All the patients provided written informed consent before enrollment in the study.

None of the patients in any of the cohorts received preoperative treatment. Preoperative radiological findings were based on the last computed tomography (CT) scan before surgery. Histological diagnosis of surgically resected specimens was made in accordance with the World Health Organization classification (Hruban et al. 2019). All the tumors were classified according to the Tumor-Node-Metastasis Classification of the International Union Against Cancer (Brierley et al. 2017). The clinicopathological parameters of patients belonging to each cohort are summarized in Table S1.

After surgery, PDAC patients usually attended for follow-up visits with laboratory evaluations every 3 months and CT scans every 6 months for the first 2 years. The patients in the initial cohort did not receive any adjuvant therapy, whereas an oral fluoropyrimidine derivative, S-1, was prescribed to some of the validation cohort patients after surgery. Recurrence was diagnosed by clinicians on the basis of physical examination and imaging modalities such as CT, magnetic resonance imaging, or positron emission tomography, and sometimes confirmed pathologically by biopsy. The follow-up period for the initial and validation cohorts ranged from 92 to 4578 days (mean, 642.5 days) and from 87 to 909 days (mean, 418.5 days), respectively, after surgery.

### Tissue preparation and bisulfite modification

Tissue specimens in the initial cohort (17 C, 34 N and 82 T samples) were frozen immediately after surgery and preserved in tanks of liquid nitrogen at the National Cancer Center Biobank, Tokyo, Japan, and those in the validation cohort (36 T samples) were fixed with 10% neutral-buffered formalin and embedded in paraffin, all in accordance with the Japanese Society of Pathology Guidelines for the Handling of Pathological Tissue Samples for Genomic Research (Kanai et al. 2018). From fresh-frozen tissue samples in the initial cohort, high-molecular-weight DNA was extracted using phenol–chloroform followed by dialysis. We performed microdissection of each sample of formalin-fixed paraffin-embedded tissue in the validation cohort: areas showing a tumor cell content of more than 80% were dissected using a toothpick under a microscope, avoiding contamination with stromal cells such as infiltrating inflammatory cells and fibroblasts, and non-cancerous epithelial cells. Subsequently, the genomic DNA was extracted from the microdissected specimens using a GeneRead FFPE Kit (QIAGEN GmbH, Hilden, Germany), and 500 and 100 ng of genomic DNA from fresh-frozen tissue samples and microdissected formalin-fixed paraffin-embedded tissue samples, respectively, were subjected to bisulfite treatment using an EpiTect Bisulfite Kit (QIAGEN), in accordance with the manufacturer’s protocol.

### Infinium assay

After bisulfite treatment, the DNA methylation status at 485,764 probe sites was examined at single-CpG resolution using the Infinium HumanMethylation450K BeadChip (Illumina, San Diego, CA), in accordance with the manufacturer’s instructions. Hybridization fluorescence signals were read with an iScan reader (Illumina). The data were assembled using GenomeStudio methylation software (Illumina). At each CpG site, the ratio of the fluorescent signal was measured using a methylated probe relative to the sum of the methylated and unmethylated probes, i.e., the so-called β-value, which ranges from 0.00 to 1.00, representing the fully unmethylated and fully methylated values for an individual CpG site, respectively. The results of Infinium assay have been deposited in the Gene Expression Omnibus (GEO) database (https://www.ncbi.nlm.nih.gov/geo/) (accession number: GSE155353).

### DNA methylation quantification by pyrosequencing

The PCR and sequencing primers were designed using PSQ Assay Design Software Version 1.0.6 (Biotage, Uppsala, Sweden). To overcome any PCR bias, we optimized the PCR conditions: 0%, 50%, and 100% of the fully methylated control DNA (Epitect methylated human control DNA, QIAGEN) were used as a template to test the linearity of the protocol, as described previously (Fujimoto et al. 2020; Nagashio et al. 2011). The optimized PCR conditions, i.e., PCR cycle and DNA polymerase, for each primer set are summarized in Table S2. The biotinylated PCR product was captured on streptavidin-coated beads. Quantitative sequencing was performed on a PyroMark Q24 (QIAGEN) using the Pyro Gold Reagents (QIAGEN) in accordance with the manufacturer’s protocol. The experiment was conducted in duplicate and the mean DNA methylation level was used as a quantitative value for each sample. When the difference between two measurements was more than 10%, the mean DNA methylation level determined from triplicate experiments was used as a quantitative value.

### Immunohistochemistry

Five-micrometer-thick sections of formalin-fixed paraffin-embedded tissue specimens in the validation cohort were deparaffinized and dehydrated. All sections were incubated with anti-human CDK14 rabbit polyclonal antibody (HPA015267, Atlas Antibodies AB, Bromma, Sweden; dilution 1:50). Before incubation, the sections were heated for 40 min at 70 °C in a water bath using citrate buffer at pH 6 (Genostaff Co., Ltd., Tokyo, Japan) for antigen retrieval. Endogenous peroxidase activity was blocked by 0.3% hydrogen peroxide in methanol. Non-specific reactions were blocked with 2.5% normal horse serum (Vector Laboratories, Inc., Burlingame, CA). The primary antibody incubation was conducted at 4 °C overnight, followed by incubation with VECTOR ImmPRESS HRP reagent anti-rabbit IgG (MP-7401, Vector Laboratories, Inc.) at room temperature for 30 min. 3.3′-Diaminobenzidine tetrahydrochloride was used as the chromogen. All sections were counterstained with hematoxylin.

Strong CDK14 immunoreactivity was detected in the nucleus in a proportion of cancer cells, whereas slight immunoreactivity was observed in the cytoplasm of most cancer cells. Therefore, distinct nuclear immunoreactivity was considered as positive and the incidence of nuclear CDK14 immunoreactivity was quantitatively evaluated. For each sample in the validation cohort (*n* = 36), 5 areas including at least 200 (not more than 300) cancer cells were randomly counted. The incidence of positive immunoreactivity in each area was expressed as a percentage of all the cells counted. The incidence in each sample (*n* = 36) was calculated by taking the average value for all of the 5 areas.

### Statistics

In the Infinium assay, the call proportions (*P* values for detection of signals above the background < 0.01) for 809 probes in all of the 133 tissue samples of the initial cohort were less than 90%. Since such a low proportion may have been attributable to polymorphism at the probe CpG sites, these 809 probes were excluded from the present assay, as described previously (Kuramoto et al. 2017; Ohara et al. 2017). In addition, all 11,648 probes on chromosomes X and Y were excluded to avoid any gender-specific methylation bias, leaving a final total of 473,457 autosomal CpG sites.

The DNA methylation profiles of the initial cohort (17 C, 34 N, and 82 T samples) were analyzed using principal component analysis (PCA). Hierarchical clustering (Ward’s linkage using Euclidean distances) was performed based on Infinium data for 82 T samples in the initial cohort. Infinium probes showing significant differences in DNA methylation levels between sample groups were defined by Welch’s *t* test. The associations between clinicopathological parameters and DNA methylation alterations were evaluated using Fisher’s exact test. A receiver-operating characteristic (ROC) curve was generated for discriminating the ER group from the non-ER group, and the Youden index for each probe was used as a cut-off value for prediction of ER. All statistical analyses were performed using the statistical program RStudio (RStudio Inc., Boston, MA) (https://www.rstudio.com), the R software package (R Foundation for Statistical Computing) (https://www.r-project.org), and JMP 12 (SAS Institute Inc., Cary, NC).

## Results

### DNA methylation profiles based on Infinium assay

**Fig. 1:**
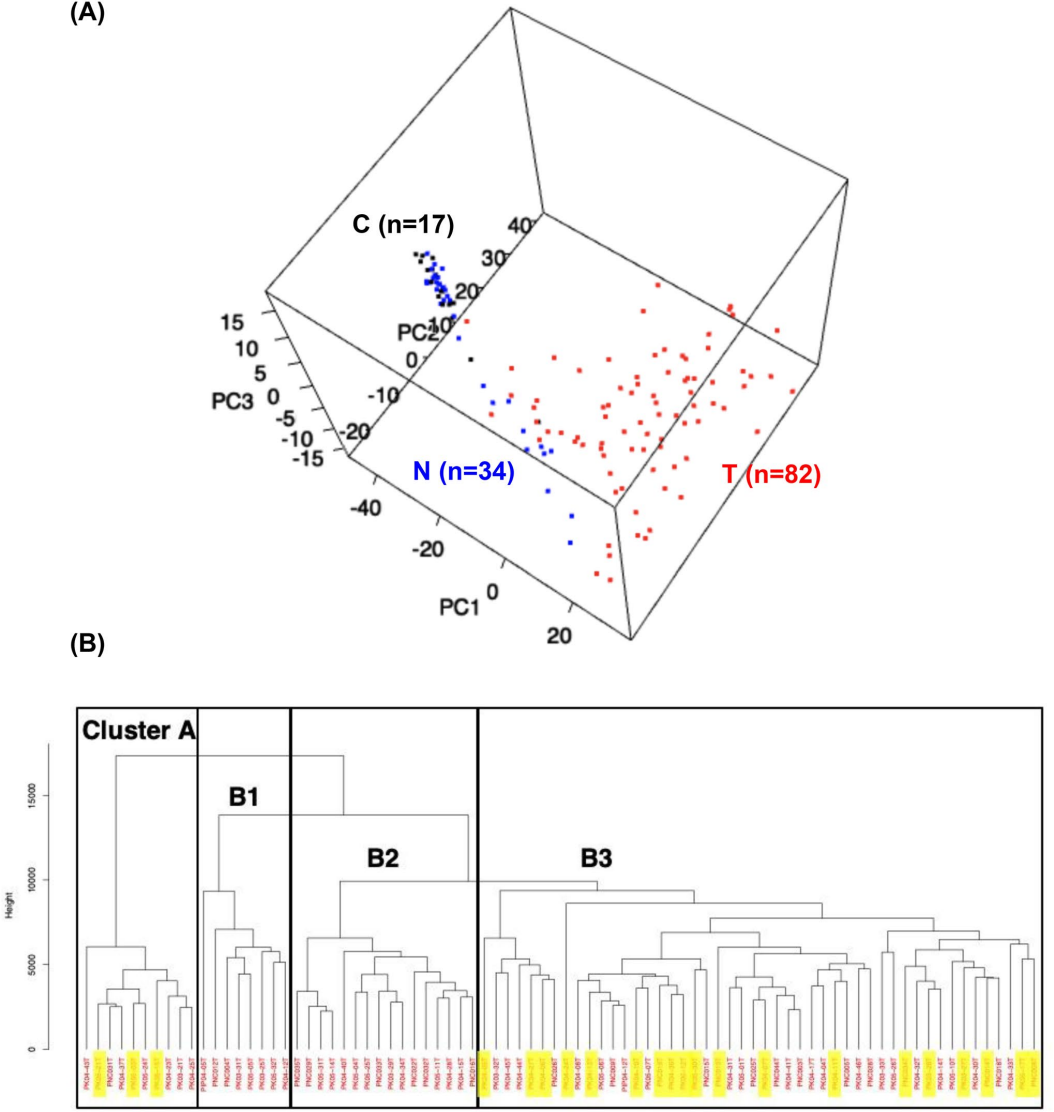
DNA methylation profiles based on Infinium assay in the initial cohort. **a** Principal component analysis of all 133 samples (17 C, 34 N and 82 T samples) using the 91,907 probes that were aberrantly methylated in the T samples (*P* value < 0.05 by Welch’s *t* test with Bonferroni correction and Δβ_T-C_ value > 0.1 or < − 0.1) compared to the C samples. PC, principal component. **b** Hierarchical clustering (Ward’s linkage using Euclidean distances) in 82 T samples using the 66,789 probes that were aberrantly methylated in T samples (*P* value < 0.05 by Welch’s *t* test with Bonferroni correction and Δβ_T-N_ value > 0.1 or < − 0.1) compared to the N samples. T samples were clustered into four subclasses: Clusters A (*n* = 10), B1 (*n* = 8), B2 (*n* = 16), and B3 (*n* = 48). Patients showing early recurrence defined as relapse within 6 months after surgery are marked with yellow

The Infinium assay identified 91,907 probes that were aberrantly methylated in the 82 T samples of the initial cohort (*P* value < 0.05 by Welch’s *t* test with Bonferroni correction and Δβ_T-C_ value > 0.1 or < − 0.1) compared to the 17 C samples, indicating that DNA methylation alterations had occurred in T samples and participate in pancreatic carcinogenesis. Among the 91,907 probes, 59,900 showed DNA hypermethylation in T samples relative to C samples, whereas 32,007 probes showed DNA hypomethylation in T samples relative to C samples. PCA of 17 C samples, 34 N samples, and 82 T samples of the initial cohort using the 91,907 probes indicated that the DNA methylation profile of N samples tended to differ from that of C samples (Fig. 1a). Furthermore, the DNA methylation profile of T samples clearly differed from that of both C and N samples (Fig. 1a).

Subsequently, we focused on the N-to-T transition stage during carcinogenesis and identified 66,789 probes that were aberrantly methylated in the 82 T samples of the initial cohort (*P* value < 0.05 by Welch’s *t* test with Bonferroni correction and Δβ_T-N_ value > 0.1 or < − 0.1) compared to the 34 N samples. Among the 66,789 probes, 43,184 showed DNA hypermethylation in T samples relative to N samples, whereas 23,605 probes showed DNA hypomethylation in T samples relative to N samples. Using the 66,789 probes, hierarchical clustering (Ward’s linkage using Euclidean distances) of 82 T samples was performed (Fig. 1b): 82 T samples were clustered into four subclasses: Clusters A (*n* = 10), B1 (*n* = 8), B2 (*n* = 16), and B3 (*n* = 48). The clinicopathological parameters of the epigenetic clusters are summarized in Table S3A. Although Table S3A shows a significant association between epigenetic clustering and lymphovascular invasion, a low incidence of lymphovascular invasion was restricted to the smallest Cluster B1 (*n* = 8) and such an association appeared to lack any clinical impact. On the other hand, Table S3A suggested that Clusters A and B3 shared a tendency for a larger tumor size. Therefore, we decided to combine clusters with similar tendencies into Clusters A and B3 and Clusters B1 and B2 to highlight the differences in their clinicopathological parameters (Table S3B). PDAC tumors belonging to Clusters A and B3 were significantly larger than those belonging to Clusters B1 and B2 (*P* = 0.030). Moreover, when ER was defined as relapse within 6 months after surgery, all 22 patients showing ER were included in Cluster A or B3, as shown in Fig. 1b: the incidence of ER in Cluster A or B3 (37.9%) was significantly higher than that in Clusters B1 and B2 (0%, *P* = 0.0002).

### Identification of marker CpG sites for discrimination of the ER group

**Fig. 2:**
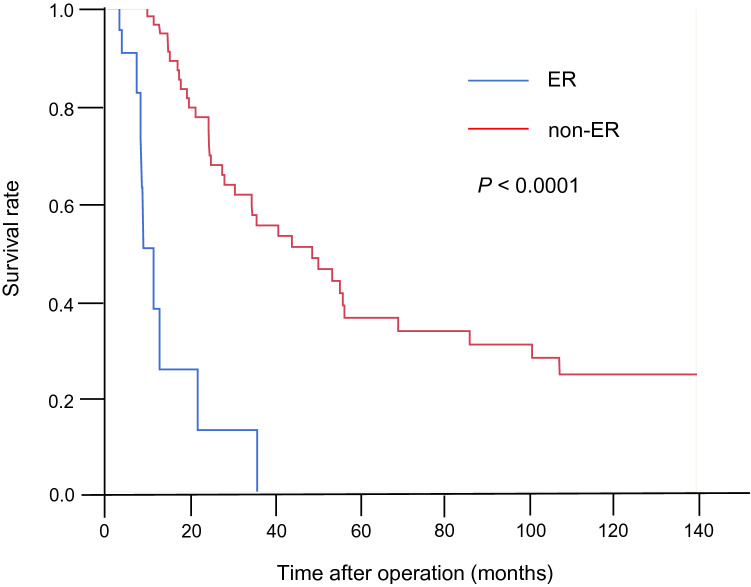
Overall survival of patients in the initial cohort. When early recurrence (ER) was defined as relapse within 6 months after surgery, patients belonging to the ER group (*n* = 22) showed a poorer outcome than patients belonging to the non-ER group (*n* = 60) (*P* < 0.0001 by Log-rank test)

At the time of last follow-up in the initial cohort (*n* = 82), 71 patients (86.6%) had suffered disease recurrence after a median recurrence-free survival (RFS) period of 12.4 months. There were no significant differences in any of the clinicopathological parameters reflecting tumor aggressiveness between the ER group (*n* = 22) and the non-ER group who suffered recurrence 6 months or later after surgery (*n* = 60) (Table S4), indicating that the risk of ER cannot be predicted based on such parameters, including the level of the widely used serum tumor marker CA19-9. Median overall survival (OS) for the entire cohort was 35.6 months. On the other hand, OS for the ER group was 11.2 months, whereas that for the non-ER group was 48.9 months (Fig. 2, *P* < 0.0001 by Log-rank test), indicating the importance of ER prediction for deciding the follow-up strategy for patients with PDAC to improve their outcome.

**Fig. 3:**
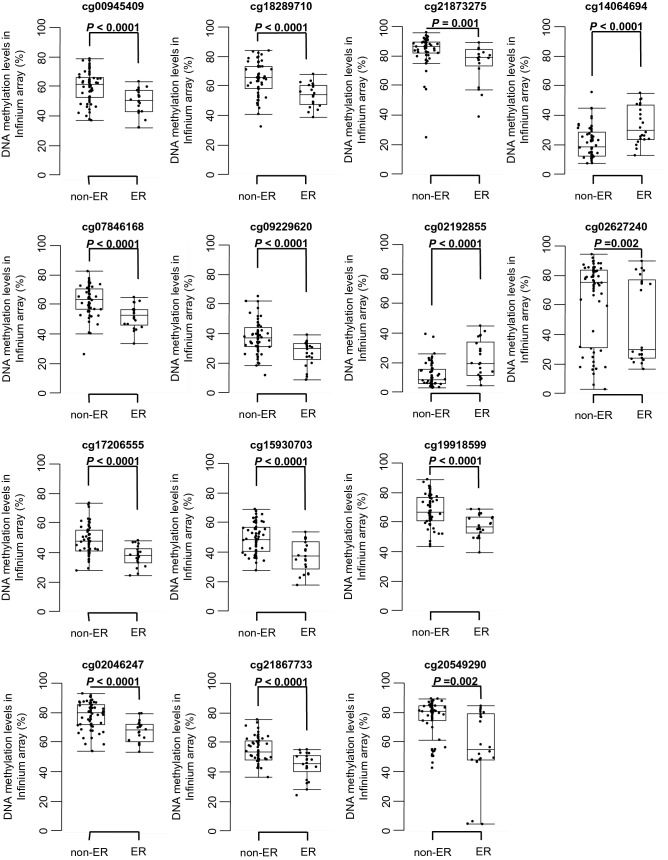
DNA methylation levels obtained by Infinium assay of candidate marker CpG sites in tissue samples in the early recurrence (ER) group (*n* = 22) and the non-ER group (*n* = 60) in the initial cohort. Infinium probe IDs and *P* values in Welch’s *t* test are shown in each panel. DNA methylation levels for each of the Infinium probes, cg14064694 and cg02192855, in the ER group are significantly higher than those in the non-ER group. The DNA methylation levels for each of the Infinium probes, cg00945409, cg18289710, cg21873275, cg07846168, cg09229620, cg02627240, cg17206555, cg15930703, cg19918599, cg02046247, cg21867733, and cg20549290, in the ER group are significantly lower than those in the non-ER group

Since our Infinium assay indicated that DNA methylation profiles have some impact on the aggressiveness of PDAC (Fig. 1b and Table S3B), we attempted to establish diagnostic criteria for prediction of ER based on DNA methylation. Welch’s *t* test identified 134 probes showing significant differences in DNA methylation levels between the ER and non-ER groups (*P* value < 0.05 and Δβ_ER-non-ER_ value > 0.1 or < − 0.1). Next, using these 134 probe CpG sites, the ROC curves were generated to discriminate patients belonging to the ER group from patients belonging to the non-ER group. Among the 134 CpG sites, 58 showed AUC values of more than 0.7 and are summarized in Table S5. Among them, scattergrams of DNA methylation levels for the 14 representative CpG sites marked by asterisks in Table S5 are shown in Fig. 3, indicating that these 14 CpG sites could be candidate markers for discrimination of the ER group.

### Pyrosequencing for technical verification of DNA methylation levels of candidate marker CpG sites

The Infinium assay is generally considered to be a genome-wide screening method, whereas pyrosequencing is a precise method for quantification of DNA methylation at specific CpG sites. In fact, when we preliminarily established ER prediction criteria based solely on Infinium data for the marker CpG sites included in Table S5 and Fig. 3, the overall diagnostic impact was insufficient (data not shown). In addition, pyrosequencing of individual marker CpG sites is more suitable in a clinical laboratory situation. Therefore, we decided to establish our ER prediction criteria based on pyrosequencing.

Although we intended to technically verify the DNA methylation levels of the 14 candidate marker CpG sites using pyrosequencing, optimization of the PCR conditions was very difficult for pyrosequencing of 2 of the 14 CpG sites (cg21867733, cg02627240). On the other hand, the PCR conditions for pyrosequencing were successfully optimized for verification of the remaining 12 candidate marker CpG sites (cg00945409, cg07846168, cg17206555, cg02046247, cg18289710, cg09229620, cg15930703, cg21873275, cg02192855, cg19918599, cg20549290, and cg14064694) (Table S2): the linearity of the measured values and their consistency with the theoretical values for the 12 CpG sites are shown in Figure S1.

The DNA methylation levels of the 12 CpG sites were quantified using pyrosequencing in the initial cohort, and all were found to be significantly correlated with those obtained using the Infinium assay (Pearson correlation coefficient [r] ≥ 0.801 and *P* < 0.0001) (Figure S2), indicating that the Infinium data had been successfully verified by pyrosequencing and that the 12 CpG sites identified on the basis of the Infinium assay were valid prognostic markers. Table 1 summarizes the gene names, chromosomes, CpG types (islands, island shores [2000-bp regions adjacent to a CpG island], and island shelves [2000-bp regions adjacent to an island shore] based on the University of California, Santa Cruz [UCSC] genome browser [https://genome.ucsc.edu/]) and annotations (TSS1500 [from 200 bp upstream of the transcription start site [TSS] to 1500 bp upstream of it], TSS200 [from TSS to 200 bp upstream of it], 5′ UTR [untranslated region], 1st exon, 1st intron, and gene body [2nd exon and downstream] identified using the RefSeq database [http://www.ncbi.nlm.nih.gov/refseq/]) for the 12 CpG sites.

**Table 1:** Potential marker CpG sites discriminating the early recurrence (ER) group from the non-ER group based on Infinium assay, for which data had been verified using pyrosequencing in the initial cohort

Probe ID^a^	Chromosome	Gene symbol^b^	CpG type^c^	Gene region^d^
cg00945409	10	*ZMIZ1-AS1*	S_Shelf	Gene body
cg07846168	13	*GPC6*	Open sea	Gene body
cg17206555	7	*CDK14*	S_Shore	First intron
cg02046247	12	*NA*	Open sea	Intergenic region
cg18289710	11	*PDGFD*	Open sea	Gene body
cg09229620	3	*NLGN1*	S_Shore	5′ UTR
cg15930703	2	*DOCK10*	Open sea	Gene body
cg21867733	12	*NCOR2*	Open sea	5′ UTR
cg21873275	1	*NBPF25P*	Open sea	Gene body
cg02192855	6	*HIST1H2BI*	N_Shore	TSS200
cg19918599	3	*NA*	Open sea	Intergenic region
cg20549290	7	*GIMAP4*	Open sea	TSS1500
cg14064694	6	*HIST1H2BI*	Island	First exon
cg02627240	7	*TPK1*	Open sea	Gene body

^a^Probe IDs for the Infinium HumanMethylation450 BeadChip (Illumina)

^b^NA: not annotated (located within intergenic regions)

^c^CpG islands, island shores (2000-bp regions adjacent to a CpG island), and island shelves (2000-bp regions adjacent to an island shore) are identified based on the University of California, Santa Cruz (UCSC) genome browser (https://genome.ucsc.edu/)

^d^TSS1500 (from 200 bp upstream of the transcription start site [TSS] to 1500 bp upstream of it), TSS200 (from TSS to 200 bp upstream of it), 5′ UTR (untranslated region), first exon, first intron, and gene body (second exon and downstream) are identified based on the RefSeq database (http://www.ncbi.nlm.nih.gov/refseq/)

Table 1 included a CpG island, island shores, and a shelf around TSS, i.e., TSS200, TSS1500, 1st exon, and 1st intron, which are considered to be critical for regulating the expression of specific genes. On the other hand, not a few surrogate marker CpG sites were identified even in open sea regions in the gene bodies (Table 1), suggesting that CpG sites regulating the expression of tumor-related genes potentially participating in tumor aggressiveness, including ER, and those that may not participate in such gene expression regulation but are probably included in the domains that are controlled in parallel with the level of DNA methylation of such tumor-related genes, could be used as ER predictors.

### Establishment of criteria for prediction of ER

To establish criteria for prediction of ER using each of the 12 marker CpG sites, ROC curves were generated using the pyrosequencing data for discriminating patients belonging to the ER group from those belonging to the non-ER group (Figure S3), and Youden indices were set as cut-off values for each CpG site. Even when pyrosequencing data were used, AUC values of > 0.7 were again obtained for each of the 12 exact Infinium probe CpG sites (Table 2).

**Table 2:** DNA methylation diagnostics for the early recurrence (ER) group based on pyrosequencing data

Probe ID^a^	Position^b^	DNA methylation status^c^	AUC	Cut-off value (%)	Initial cohort		Validation cohort	
					Sensitivity (%)^d^	Specificity (%)^e^	Sensitivity (%)^d^	Specificity (%)^e^
cg00945409	1	ER < non-ER	0.778	58.6	90.9	60.0	66.7	44.4
	2		0.764	71.6	86.4	68.3	22.2	88.9
cg07846168	1	ER < non-ER	0.791	61.3	95.5	56.7	NA	NA
cg17206555	1	ER < non-ER	0.811	23.5	86.4	71.7	66.7	77.8
	2		0.760	18.7	86.4	65.0	55.6	74.1
cg02046247	1	ER < non-ER	0.775	79.0	90.9	60.0	88.9	63.0
	2		0.739	92.0	86.4	53.3	88.9	7.41
cg18289710	1	ER < non-ER	0.759	61.6	72.7	70.0	77.8	22.2
cg09229620	2	ER < non-ER	0.708	41.7	77.3	56.7	NA	NA
	3		0.715	62.8	77.3	56.7	NA	NA
cg15930703	1	ER < non-ER	0.753	42.9	100	40.0	88.9	14.8
	2		0.730	28.9	63.6	80.0	55.6	40.7
cg21873275	1	ER < non-ER	0.744	77.7	72.7	68.3	44.4	74.1
cg02192855	1	ER > non-ER	0.720	13.1	50.0	93.3	22.2	63.0
	3		0.773	7.32	81.8	60.0	44.4	37.0
cg19918599	1	ER < non-ER	0.759	77.9	86.4	58.3	77.8	55.6
	2		0.721	70.2	77.3	60.0	77.8	59.3
cg20549290	1	ER < non-ER	0.777	84.1	72.7	75.0	NA	NA
	2		0.739	82.0	77.3	61.7	NA	NA
cg14064694	2	ER > non-ER	0.714	19.0	54.6	81.7	33.3	51.9
	3		0.759	9.99	95.5	45.0	77.8	29.6
	4		0.710	34.4	40.9	95.0	11.1	77.8

*AUC* area under the curve value obtained by receiver-operating characteristic curve analysis, *NA* not analyzed due to PCR failure

^a^Probe IDs for the Infinium HumanMethylation450 BeadChip (Illumina)

^b^Infinium probe CpG sites and their neighboring CpG sites which were analyzed using pyrosequencing and numbered as shown in Table S2. Exact Infinium probe CpG sites are underlined

^c^“ER < non-ER”, when the DNA methylation level of the sample was lower than the cut-off value, the sample was diagnosed as belonging to the ER group; “ER > non-ER”, when the DNA methylation level of the sample was higher than the cut-off value, the sample was diagnosed as belonging to the ER group

^d^Sensitivity is defined as the ratio of the number of tissue samples diagnosed as belonging to the ER group based on the criteria relative to the exact number of the patients belonging to the ER group

^e^Specificity is defined as the ratio of the number of tissue samples not diagnosed as belonging to the ER group using the criteria employed, relative to the exact number of patients belonging to the non-ER group

When neighboring CpG sites other than exact Infinium probe CpG sites are located within amplicons for pyrosequencing, the DNA methylation levels of such neighboring sites are simultaneously quantified by pyrosequencing. For example, in addition to the exact Infinium probe cg00945409 site (position 1), DNA methylation data for a neighboring CpG site (position 2) were obtained, as shown in Table S2. Among these 13 neighboring CpG loci, 10 also showed AUC values of > 0.7 for discriminating the ER group (Figure S3) and are included in Table 2. Using each of the 22 diagnostic criteria (12 exact Infinium probe CpG sites and their 10 neighboring CpG sites), the sensitivity for diagnosis of patients showing ER (*n* = 22) in the initial cohort patients overall (*n* = 82) ranged from 40.9 to 100%, and the specificity from 40.0 to 95.0% (Table 2).

### Confirmation of criteria reliability using the validation cohort

At the time of the last follow-up of the validation cohort (*n* = 36), 20 (55.6%) patients had suffered recurrence after a median RFS of 16.3 months. There were nine patients (25.0%) in the ER group: the incidence of ER in the validation cohort did not differ significantly from that in the initial cohort. To validate the reliability of our criteria for diagnosis of ER, pyrosequencing was performed in the validation cohort using the cut-off values, as shown in Table 2. For three candidate CpG sites (cg07846168, cg09229620, and cg20549290), sufficient PCR products were not obtained. Finally, it was confirmed that both the sensitivity and specificity at three marker CpG sites—cg17206555 (position 1), cg02046247 (position 1), and 19,918,599 (position 2)—were more than 59% in the validation cohort.

Subsequently, a diagnostic panel was established by combining these three marker CpG sites, as shown in Table 3. When two or more CpG sites satisfied the criteria, as shown in Table 2, sufficient sensitivity and specificity for identification of the ER group patients were obtained in both the initial (81.8% and 71.7%) and validation (88.9% and 70.4%) cohorts, respectively (Table 3). If higher sensitivity is needed before intensive screening of recurrent and/or metastatic lesions, positivity for only one of three CpG sites may be adequate. On the other hand, if higher specificity is needed before adjuvant therapy, positivity for all three CpG sites may be adequate as criteria.

**Table 3:** The criteria combining the validated marker CpG sites, cg17206555 (position 1), cg02046247 (position 1), and cg19918599 (position 2), for prediction of early recurrence (ER) of pancreatic ductal adenocarcinoma

Number of CpG sites satisfying the criteria shown in Table 2	Initial cohort		Validation cohort	
	Sensitivity^a^ (%)	Specificity^b^ (%)	Sensitivity^a^ (%)	Specificity^b^ (%)
One or more	100	46.7	88.9	25.3
Two or three	81.8	71.7	88.9	70.4
Three	54.6	86.7	55.6	88.9

^a^Sensitivity is defined as the ratio of the number of tissue samples diagnosed as belonging to the ER group based on the criteria relative to the exact number of the patients belonging to the ER group

^b^Specificity is defined as the ratio of the number of tissue samples not diagnosed as belonging to the ER group using the criteria employed, relative to the exact number of patients belonging to the non-ER group

### Immunohistochemistry for CDK14 expression in PDACs

Since DNA hypomethylation of the *CDK14* (*cyclin-dependent kinase 14*) gene was an excellent marker for ER prediction (Table 3), CDK14 protein expression was examined by immunohistochemistry. Representative photos of CDK14-negative and CDK14-positive areas of PDACs are shown in Figure S4A. As shown in Figure S4B, the incidence of nuclear immunoreactivity for CDK14 in PDACs belonging to the ER group (*n* = 9) is significantly higher than that in PDACs belonging to the non-ER group (*n* = 27) in the validation cohort (*P* = 0.0020), indicating the possibility that the level of nuclear CDK14 expression could be used as an ER predictor.

## Discussion

Based on the present PCA by Infinium assay, the DNA methylation profile of N samples, which were obtained from patients with PDAC and may already have been exposed to carcinogenetic factors, tended to differ from that of C samples (Fig. 1a). Not a few N samples showed chronic pancreatitis, which is widely considered to be one of the precancerous conditions for PDAC (Hassan et al. 2007). Therefore, the differences in DNA methylation profiles between C and N samples may have been at least partly attributable to differences in their cellular components: N samples would have contained more infiltrating lymphocytes and fibroblasts in the background of chronic inflammation. On the other hand, our previous meticulous microdissection technique and immunohistochemistry have revealed that peripheral pancreatic duct epithelial cells, i.e., the origin of ductal adenocarcinoma (Hruban et al. 1997), and not surrounding lymphocytes or fibroblasts, actually show cumulative DNA methylation abnormalities of tumor-related genes associated with aberrant expression of DNMT1 at the precancerous N stages (Peng et al. 2005, 2006). Analogous with the results of these previous studies, the differences in the DNA methylation profiles of N samples relative to C samples in the present study may have been at least partly induced in peripheral pancreatic duct epithelial cells themselves by precancerous conditions such as chronic pancreatitis. Such participation of DNA methylation alterations even in the early and precancerous stages of multistage carcinogenesis is consistent with the results of our previous Infinium assay using many samples of cancerous tissue from various organs (Arai et al. 2009, 2012; Sato et al. 2014; Yamanoi et al. 2015).

The DNA methylation profile of T samples clearly differed from that of both C and N samples (Fig. 1a). Since, in the initial cohort, Infinium assay was performed using bulk frozen tissue samples, at least some of the differences in DNA methylation profiles between T and other samples may again have been attributable to differences in their cellular components: C and N samples contain not only peripheral pancreatic ducts, the origin of ductal adenocarcinoma (Hruban et al. 1997), but also acinar cells and islet cells. On the other hand, on the PCA scattergram (Fig. 1a), N samples were located nearer to T samples than to C samples. This finding is again consistent with the previous results from various organs where DNA methylation alterations at the precancerous stage are inherited by or strengthened in the tumorous tissue themselves (Arai et al. 2009, 2012; Sato et al. 2014; Yamanoi et al. 2015).

When we focused on the DNA methylation profiles of T samples themselves, it was possible to subclassify pancreatic cancer patients into epigenomic Clusters A and B3 vs Clusters B1 and B2, which were significantly correlated with the clinicopathological aggressiveness of PDAC, i.e., larger tumor size and early recurrence, indicating that DNA methylation abnormalities at least partly determine the malignant potential of cancers, as has been observed in many other organs (Arai et al. 2009, 2012; Sato et al. 2014; Yamanoi et al. 2015). This finding motivated us to perform prognostication of patients with PDAC based on their DNA methylation profiles. Prediction of ER risk after surgery is critical for improving the outcome of PDAC. Although previous studies that separately defined ER as occurring at 6 (Matsumoto et al. 2015), 8 (Nishio et al. 2017), or 12 (Groot et al. 2019) months after surgery, since the present study revealed that patients suffering recurrence within 6 months after curative resection clearly showed poorer survival (Fig. 2), we defined ER as relapse occurring within 6 months after surgery. Although it was not possible to predict ER on the basis of clinicopathological parameters (Table S4), we successfully identified DNA methylation biomarkers for prediction of ER based on technical verification by pyrosequencing and biological validation using the validation cohort. Most previous studies have based prognostication of patients with PDAC on clinicopathological findings (Matsumoto et al. 2015) or perioperative serum markers (Sugiura et al. 2012), and this approach has not yet achieved sufficient predictive accuracy. To our knowledge, this study is the first reported to have employed DNA methylation biomarkers for ER prediction. Unlike alterations of mRNA and protein expression, which can be easily affected by the microenvironment of cancer cells, DNA methylation alterations are stably preserved on DNA double strands by covalent bonds. Therefore, alterations of DNA methylation would potentially be optimal prognostic indicators for affected patients.

Here, we used formalin-fixed paraffin-embedded surgically resected materials in the validation study after genome-wide screening, because usage of such materials is very feasible in a clinical setting. However, the prognostic impact of some candidate marker CpG sites based on Infinium assay in the initial cohort failed to be verified in the validation cohort. This discrepancy of DNA methylation data between formalin-fixed paraffin-embedded tissue and fresh-frozen tissue was consistent with a previous report by Wen et al. (2017), who suggested that the reasons for such discrepancy might include formaldehyde-induced cross-links, DNA fragmentation, and deamination of cytosine bases. Another reason may be differences in the tumor cell content. Since bulk tissue used in the initial screening had a generally low tumor cell content, the Infinium assay would have potentially highlighted CpG sites showing DNA methylation abnormalities in fibroblasts and endothelial cells in the stroma of aggressive cancers with a risk of ER. It is known that inflammatory cytokines can affect the DNA methylation status of cells (Mishra 2020). Since infiltrating inflammatory cells secrete inflammatory cytokines, the DNA methylation status of both cancer cells and stromal cells of PDACs derived from a background of chronic pancreatitis might be regulated by the cytokine-rich tumor microenvironment. Various growth factors are known to be produced by both cancer cells and stromal cells, and potentiate cancer cell proliferation and/or invasiveness in an autocrine and paracrine manner. Therefore, it is feasible that the DNA methylation status of both cancer cells and stromal cells is altered, and that such alteration might determine tumor aggressiveness via alterations in the expression levels of such growth factors.

On the other hand, before the validation study, we microscopically dissected areas showing a tumor cell content of more than 80% from formalin-fixed paraffin-embedded specimens. Therefore, CpG sites reflecting abnormalities of only non-cancerous stromal cells, such as fibroblasts and endothelial cells, and not those of tumor cells were not verified in the validation study. Finally, after the validation study, we were able to successfully identify CpG sites that could be applicable as biomarkers regardless of tumor cell content, even using formalin-fixed paraffin-embedded specimens. Table 3 indicates that it would be possible to predict ER by quantifying only three validated marker CpG sites. Such a small number of quantification targets suggested that this approach would be feasible as a laboratory examination in a clinical setting, as well as being applicable to formalin-fixed paraffin-embedded surgically resected materials.

We additionally tried to identify prognostic markers in Cluster A and Cluster B3 individually. In Cluster A, 484 CpG sites showing significant differences in DNA methylation levels between the ER and non-ER groups (*P* value < 0.05 and Δβ_ER-non-ER_ value > 0.1 or < − 0.1) and AUC values of > 0.7 were identified. In addition, 143 marker CpG sites were identified in Cluster B3. However, the sensitivity and specificity for all samples in the initial cohort were very low (data not shown), indicating the possibility of overfitting.

Table 3 shows that a CpG site of DNA hypomethylation of the *CDK14* gene was an excellent marker for ER prediction. Since the marker CpG site (position 1 of cg17206555) is located within the CpG island shore around the TSS (Table 1), such DNA hypomethylation would potentially result in overexpression of the gene in aggressive PDAC. Indeed, a significant inverse correlation between DNA methylation and mRNA expression levels (*r* = − 0.442, *P* < 0.0001) was confirmed based on data for samples of cancerous and non-cancerous pancreatic tissue (*n* = 182) deposited in the TCGA database (https://www.cancer.gov/about-nci/organization/ccg/research/structural-genomics/tcga) (Figure S5). In fact, our immunohistochemical examination revealed a significantly higher incidence of nuclear CDK14 immunoreactivity in PDACs in the ER group than that in the non-ER group (Figure S4). Even though sufficient sensitivity and specificity cannot be obtained solely by immunohistochemical examination, in a clinical setting, such examination using formalin-fixed paraffin-embedded tissue specimens of surgically resected material would be an auxiliary procedure for ER risk diagnosis based on quantification of DNA methylation.

CDK14 reportedly participates in the proliferation and invasion of many tumor cells, including those of breast cancer (Imawari et al. 2018), glioma (Fan et al. 2015), hepatocellular carcinoma (Tu et al. 2019), and ovarian cancer (Ou-Yang et al. 2017). In pancreatic cancers, *CDK14* has been revealed as a hub gene in the interaction network including the Wnt/β-catenin pathway and phosphoinositide 3-kinase/Akt signaling pathway, and its increased expression is associated with poorer overall patient survival (Yuan et al. 2019). Although there are no published data to suggest that *CDK 14* gene expression is regulated by DNA methylation, it is feasible that DNA hypomethylation might affect the risk of ER in PDAC through overexpression of *CDK 14*.

On the other hand, other marker CpG sites, position 1 of cg02046247 and position 2 of cg19918599, included in Table 3 are located outside CpG islands (open sea regions) in intergenic regions and would not participate in regulating the expression of specific genes. This is consistent with the results of our previous studies, indicating that even DNA methylation alterations at CpG sites not involved in the expression of functionally important genes would be potentially excellent surrogate markers for cancer diagnostics (Fujimoto et al. 2020; Nagashio et al. 2011). In general, most 5-methylcytosine residues seem to be controlled in a coordinated manner with their neighbors, rather than being independent, resulting in a domain wherein all CpG sites show largely similar methylation levels (Yokoyama et al. 2015). During carcinogenesis, the DNA methylation status of neighboring CpG sites included in the same domain is frequently altered *en bloc* (Johnstone et al. 2020). If such a domain includes important tumor-related genes whose function determines the ER of PDACs, it is feasible that the DNA methylation levels of neighboring CpG sites in the same domain could become surrogate markers for ER, even though they are not directly involved in regulation of specific genes.

In summary, we have identified potential biomarker CpG sites and established criteria that would be applicable to formalin-fixed paraffin-embedded tissue samples, and also to tumors both with and without an abundant cancer stroma consisting of infiltrating lymphocytes, fibroblasts, and other stromal cells, for prediction of ER of PDAC. Even though a large-scale prospective validation study is of course needed, such prognostication based on DNA methylation diagnostics may provide a breakthrough for personalized treatment of patients with aggressive PDAC. In addition, we have developed a system for quantification of DNA methylation involving high-performance liquid chromatography [HPLC], which is suitable for diagnosis of clinical samples containing various cell linages (Yotani et al. 2018). Using such appropriate diagnostic approaches, we expect that our markers would be applicable for prognostication using samples of pancreatic juice and blood including circulating tumors cells or cell-free DNA samples.

## Supplementary Information

Below is the link to the electronic supplementary material.

Supplementary file1 (PDF 230 KB)

Supplementary file2 (PDF 1462 KB)

## Data Availability

The Infinium assay data have been deposited in the Gene Expression Omnibus (GEO) database (https://www.ncbi.nlm.nih.gov/geo/) (accession number: GSE155353).

## Abbreviations

AUC: Area under the curve
BAC: Bacterial artificial chromosome
C: Normal control pancreatic tissue
CA19-9: Carbohydrate antigen 19-9
CT: Computed tomography
DNMT: DNA methyltransferase
ER: Early recurrence
GEO: Gene Expression Omnibus
N: Non-cancerous pancreatic tissue
ICGC: International Cancer Genome Consortium
OS: Overall survival
PCA: Principal component analysis
PDAC: Pancreatic ductal adenocarcinoma
ROC: Receiver-operating characteristic
RFS: Recurrence-free survival
T: Cancerous tissue
TCGA: The Cancer Genome Atlas
TSS: Transcription start site
