# Supplementary material for: Clinicopathological impacts of DNA methylation alterations on pancreatic ductal adenocarcinoma: prediction of early recurrence based on genome-wide DNA methylation profiling
Source: J Cancer Res Clin Oncol. 2021 Feb 26;147(5):1341–54. doi: 10.1007/s00432-021-03541-6 (PMC8021514; doi:10.1007/s00432-021-03541-6)
Supplement: Supplementary file 1 — Supplementary file1 (PDF 230 KB) [file 432_2021_3541_MOESM1_ESM.pdf]

Supplementary Table S1 for Review. DNA methylation diagnostics for the early recurrence (ER) group using marker CpG sites individually identified in Cluster B3 and Cluster A.

(A) Marker CpG sites identified in Cluster B3.

Target ID[a]	AUC[b]	Cutoff value (%)[c]	Sensitivity (%)[d]	Specificity (%)[e]
cg20012308	0.871	51.1	13.6	25.0
cg14064694	0.851	20.0	18.2	36.7
cg02192855	0.846	10.6	18.2	41.7
cg02677286	0.840	89.2	27.3	16.7
cg03758514	0.838	21.7	54.5	5.0
cg02627240	0.831	82.2	18.2	40.0
cg24881205	0.831	28.9	27.3	31.7
cg07148296	0.822	26.8	45.5	21.7
cg02897005	0.820	17.5	27.3	33.3
cg19851715	0.809	13.3	45.5	16.7
cg11459603	0.808	12.3	9.1	53.3
cg05502360	0.804	79.5	13.6	41.7
cg18465515	0.802	21.5	59.1	3.3
cg12569880	0.800	69.8	22.7	31.7
cg16448102	0.800	29.5	27.3	38.3
cg21873275	0.799	86.2	4.5	45.0
cg11744777	0.793	26.4	18.2	30.0
cg24922129	0.791	32.0	0.0	48.3
cg13372766	0.789	21.7	13.6	43.3
cg24208023	0.789	16.0	13.6	46.7
cg14506196	0.784	25.5	36.4	33.3
cg23233200	0.784	23.4	22.7	33.3
cg24012880	0.784	66.4	13.6	38.3
cg26516759	0.784	29.1	36.4	40.0
cg02177231	0.782	27.6	18.2	45.0
cg03721887	0.780	88.0	22.7	31.7
cg04464523	0.779	87.5	13.6	51.7
cg07681579	0.779	19.7	18.2	53.3
cg21392341	0.779	20.8	22.7	41.7
cg11428724	0.777	18.2	27.3	45.0
cg19246197	0.777	58.8	4.5	48.3
cg03209557	0.775	23.2	50.0	10.0
cg26075208	0.775	23.5	31.8	23.3
cg06316104	0.773	21.6	31.8	36.7
cg09324326	0.771	78.8	18.2	36.7
cg00945409	0.770	61.5	4.5	40.0
cg05299459	0.770	87.0	0.0	56.7
cg08816590	0.770	16.9	36.4	43.3
cg11243251	0.770	39.1	22.7	31.7
cg02496234	0.768	19.9	22.7	58.3
cg12438155	0.768	65.9	40.9	21.7
cg24332389	0.768	61.3	9.1	48.3
cg07270289	0.767	74.0	36.4	33.3
cg03573446	0.766	38.0	31.8	35.0
cg18784875	0.766	44.2	13.6	50.0
cg00034433	0.764	20.2	36.4	40.0
cg10080155	0.764	30.5	31.8	28.3
cg07112337	0.762	31.1	4.5	63.3
cg15659132	0.762	10.7	31.8	35.0
cg20340302	0.760	37.3	27.3	40.0
cg10690440	0.759	48.4	54.5	8.3
cg27028760	0.757	30.2	31.8	28.3

cg14460215	0.755	58.0	22.7	36.7
cg24828683	0.755	17.0	31.8	33.3
cg03004280	0.753	19.3	36.4	33.3
cg09229918	0.753	37.6	27.3	36.7
cg13469777	0.753	71.0	27.3	35.0
cg23217622	0.753	34.8	27.3	45.0
cg24080129	0.753	5.6	31.8	33.3
cg24875593	0.753	70.4	22.7	53.3
cg15679813	0.751	35.7	22.7	46.7
cg02355653	0.750	54.5	36.4	38.3
cg08071329	0.750	78.7	18.2	35.0
cg09994356	0.750	44.1	36.4	26.7
cg25561581	0.750	25.8	27.3	35.0
cg00474373	0.746	71.3	22.7	46.7
cg16646616	0.746	27.7	31.8	33.3
cg03225548	0.744	61.6	31.8	35.0
cg04550737	0.744	12.4	18.2	56.7
cg00584026	0.742	53.2	9.1	41.7
cg16991515	0.742	25.7	9.1	46.7
cg01296877	0.740	47.2	27.3	58.3
cg23663942	0.740	77.6	54.5	16.7
cg07482202	0.739	9.6	36.4	40.0
cg11403739	0.739	55.9	27.3	40.0
cg23764766	0.739	45.6	40.9	21.7
cg26822175	0.739	82.2	40.9	26.7
cg03292225	0.737	78.8	45.5	15.0
cg09908110	0.737	38.7	31.8	36.7
cg15069906	0.737	34.3	22.7	45.0
cg26727435	0.737	32.7	54.5	16.7
cg07903626	0.735	60.4	22.7	48.3
cg23622129	0.733	54.2	18.2	40.0
cg23816049	0.733	32.2	40.9	30.0
cg01031032	0.731	30.5	18.2	45.0
cg20090066	0.731	44.5	13.6	63.3
cg04998634	0.730	25.5	18.2	41.7
cg05540369	0.730	25.5	36.4	28.3
cg09938511	0.730	34.8	27.3	46.7
cg14168923	0.730	19.6	31.8	36.7
cg16126280	0.730	2.0	13.6	65.0
cg22901297	0.730	30.9	50.0	8.3
cg03127713	0.726	23.2	27.3	45.0
cg14786652	0.726	41.0	0.0	55.0
cg15529692	0.724	23.4	27.3	48.3
cg17123534	0.724	33.4	50.0	20.0
cg22777560	0.724	82.7	40.9	16.7
cg00274640	0.722	67.5	13.6	50.0
cg03045133	0.722	73.3	54.5	28.3
cg12518442	0.722	32.4	22.7	41.7
cg02885527	0.721	83.1	36.4	48.3
cg15744668	0.721	26.2	31.8	35.0
cg08292959	0.719	51.5	22.7	46.7
cg20495645	0.719	29.2	22.7	46.7
cg20568196	0.719	24.1	27.3	41.7
cg08003150	0.717	8.1	18.2	56.7
cg03832162	0.715	14.4	22.7	53.3
cg11978634	0.715	83.2	31.8	41.7
cg15365500	0.715	87.0	50.0	26.7
cg18574144	0.715	76.2	4.5	65.0

cg26056277	0.715	81.5	27.3	35.0
cg17370616	0.713	57.1	9.1	65.0
cg00033209	0.710	47.2	27.3	33.3
cg16999994	0.710	88.0	13.6	66.7
cg06637001	0.708	25.2	50.0	20.0
cg07723510	0.708	30.3	40.9	20.0
cg14548259	0.708	30.8	36.4	33.3
cg16397968	0.708	86.0	4.5	61.7
cg22836359	0.708	68.3	13.6	48.3
cg27230769	0.708	49.7	27.3	53.3
cg02146001	0.706	23.9	40.9	31.7
cg10006614	0.706	5.4	40.9	41.7
cg15885337	0.706	25.3	18.2	61.7
cg19566252	0.706	43.4	4.5	61.7
cg19595956	0.706	28.5	54.5	6.7
cg02772171	0.704	48.7	27.3	26.7
cg04367486	0.704	32.8	18.2	40.0
cg16282993	0.704	29.8	54.5	15.0
cg24028634	0.704	44.2	36.4	33.3
cg00269725	0.703	68.1	36.4	31.7
cg26999501	0.703	80.4	40.9	31.7
cg00609645	0.702	30.6	54.5	10.0
cg01012936	0.702	48.1	18.2	43.3
cg08151857	0.702	30.0	22.7	41.7
cg08198851	0.702	4.8	22.7	51.7
cg10941747	0.702	30.4	27.3	40.0
cg14776962	0.702	20.1	27.3	45.0
cg16480692	0.702	23.9	36.4	31.7
cg25976257	0.702	21.3	27.3	50.0
cg18572898	0.701	3.9	36.4	25.0
cg11456756	0.701	26.1	27.3	48.3
cg18861767	0.701	3.4	27.3	40.0
cg27567580	0.701	46.5	18.2	55.0

(B) Marker CpG sites identified in Cluster A.

Target ID[a]	AUC[b]	Cutoff value (%)[c]	Sensitivity (%)[d]	Specificity (%)[e]
cg00127167	1.000	28.7	13.6	85.0
cg00156497	1.000	65.2	54.5	30.0
cg00451054	1.000	74.3	9.1	75.0
cg00631329	1.000	20.1	40.9	61.7
cg00807464	1.000	23.0	68.2	21.7
cg00850068	1.000	17.1	31.8	71.7
cg00949635	1.000	64.0	4.5	88.3
cg01240056	1.000	25.2	9.1	70.0
cg01460436	1.000	38.2	27.3	38.3
cg02136690	1.000	39.1	0.0	85.0
cg02446475	1.000	49.7	50.0	41.7
cg02588321	1.000	24.2	31.8	60.0
cg02997295	1.000	38.8	72.7	13.3
cg03081173	1.000	56.8	50.0	45.0
cg03274456	1.000	44.9	59.1	50.0
cg03392386	1.000	65.4	40.9	58.3
cg03419058	1.000	11.0	13.6	73.3
cg03510349	1.000	13.8	13.6	76.7
cg03524755	1.000	77.5	68.2	26.7
cg03535793	1.000	38.9	18.2	76.7
cg03576863	1.000	48.9	59.1	45.0
cg03709428	1.000	51.2	72.7	30.0

cg03943025	1.000	22.0	72.7	3.3
cg04292549	1.000	32.6	59.1	60.0
cg04475027	1.000	22.8	4.5	76.7
cg04534765	1.000	22.8	0.0	88.3
cg04541077	1.000	71.1	50.0	31.7
cg05280336	1.000	54.9	63.6	35.0
cg05323879	1.000	29.3	40.9	56.7
cg05374271	1.000	46.3	36.4	56.7
cg05573767	1.000	39.2	27.3	65.0
cg05655647	1.000	23.0	27.3	26.7
cg05716726	1.000	47.2	13.6	78.3
cg05828690	1.000	19.7	50.0	40.0
cg05876246	1.000	26.9	0.0	88.3
cg06020335	1.000	87.6	4.5	83.3
cg06173319	1.000	28.7	50.0	50.0
cg06248560	1.000	20.2	13.6	63.3
cg06305758	1.000	51.6	63.6	36.7
cg06383163	1.000	38.8	13.6	76.7
cg06618134	1.000	29.5	81.8	16.7
cg06782035	1.000	28.7	9.1	81.7
cg06813297	1.000	65.7	68.2	18.3
cg06815950	1.000	54.6	63.6	8.3
cg07504040	1.000	72.2	63.6	28.3
cg07507129	1.000	81.9	68.2	3.3
cg07534843	1.000	21.2	27.3	63.3
cg07563363	1.000	35.0	22.7	58.3
cg07612827	1.000	68.9	54.5	46.7
cg07905054	1.000	29.6	18.2	51.7
cg07909498	1.000	31.0	54.5	23.3
cg08103988	1.000	41.3	72.7	8.3
cg08373528	1.000	52.1	54.5	41.7
cg08762424	1.000	53.7	59.1	38.3
cg08814020	1.000	58.9	22.7	75.0
cg08947187	1.000	74.7	22.7	68.3
cg09721047	1.000	56.9	59.1	63.3
cg10225865	1.000	44.3	77.3	20.0
cg10334703	1.000	43.6	9.1	85.0
cg10406205	1.000	27.0	59.1	25.0
cg10471574	1.000	45.1	9.1	60.0
cg10762214	1.000	55.4	22.7	63.3
cg10930366	1.000	52.6	27.3	55.0
cg11066601	1.000	86.7	86.4	5.0
cg11082691	1.000	30.8	9.1	71.7
cg11117637	1.000	76.8	40.9	53.3
cg11823511	1.000	28.4	4.5	85.0
cg11919837	1.000	64.0	31.8	61.7
cg12076350	1.000	60.7	63.6	36.7
cg12156287	1.000	56.9	45.5	46.7
cg12346967	1.000	72.0	36.4	33.3
cg12485185	1.000	26.3	9.1	75.0
cg12970830	1.000	91.2	77.3	16.7
cg13541353	1.000	43.5	59.1	45.0
cg13979884	1.000	62.1	59.1	63.3
cg14065526	1.000	6.7	81.8	10.0
cg14172108	1.000	35.4	27.3	73.3
cg14392168	1.000	64.2	9.1	81.7
cg14759043	1.000	26.0	18.2	73.3
cg14801238	1.000	75.5	68.2	35.0

cg14928378	1.000	55.3	40.9	43.3
cg14945473	1.000	14.9	27.3	55.0
cg15065340	1.000	60.0	4.5	83.3
cg15320980	1.000	65.4	54.5	33.3
cg15375321	1.000	46.4	54.5	26.7
cg15522953	1.000	57.5	13.6	78.3
cg15654458	1.000	84.6	77.3	20.0
cg15711973	1.000	46.7	50.0	41.7
cg15925143	1.000	67.4	36.4	66.7
cg16250754	1.000	25.9	0.0	88.3
cg16472389	1.000	12.9	54.5	23.3
cg16497714	1.000	59.7	0.0	85.0
cg16558994	1.000	15.6	50.0	33.3
cg16590012	1.000	39.5	27.3	30.0
cg16731240	1.000	31.8	22.7	61.7
cg16745616	1.000	18.7	13.6	76.7
cg16863829	1.000	63.4	59.1	40.0
cg17082366	1.000	44.7	40.9	45.0
cg17101296	1.000	31.4	13.6	80.0
cg17416644	1.000	52.5	31.8	53.3
cg17860133	1.000	67.9	0.0	85.0
cg17904739	1.000	22.3	13.6	78.3
cg18208842	1.000	31.3	18.2	51.7
cg18289303	1.000	88.3	22.7	48.3
cg18424208	1.000	74.4	50.0	51.7
cg18771570	1.000	33.3	22.7	51.7
cg18880384	1.000	68.4	36.4	51.7
cg18893000	1.000	41.9	45.5	45.0
cg19605788	1.000	36.8	27.3	73.3
cg19660806	1.000	59.3	40.9	33.3
cg19750824	1.000	35.1	68.2	30.0
cg20036791	1.000	29.2	50.0	43.3
cg20098848	1.000	66.7	59.1	10.0
cg20462978	1.000	48.9	77.3	13.3
cg20549290	1.000	64.5	40.9	20.0
cg21723903	1.000	33.1	22.7	75.0
cg22074858	1.000	21.1	0.0	83.3
cg23037321	1.000	33.1	4.5	88.3
cg23361828	1.000	74.2	36.4	46.7
cg23489038	1.000	25.7	18.2	58.3
cg23489384	1.000	2.2	59.1	25.0
cg23622369	1.000	19.6	63.6	38.3
cg24037715	1.000	65.3	59.1	3.3
cg24144083	1.000	21.8	0.0	85.0
cg24263062	1.000	59.8	13.6	71.7
cg24441899	1.000	43.3	40.9	48.3
cg24466241	1.000	32.2	22.7	73.3
cg24842815	1.000	64.5	45.5	31.7
cg24923430	1.000	44.1	40.9	35.0
cg25107000	1.000	74.7	68.2	28.3
cg25191628	1.000	17.3	0.0	85.0
cg25267487	1.000	60.5	59.1	25.0
cg25290178	1.000	82.8	27.3	60.0
cg25341313	1.000	61.5	22.7	83.3
cg25977769	1.000	3.6	63.6	36.7
cg25998181	1.000	58.3	31.8	63.3
cg26191747	1.000	57.5	45.5	51.7
cg26282792	1.000	32.9	22.7	71.7

cg26325285	1.000	32.3	81.8	6.7
cg26343358	1.000	80.8	72.7	25.0
cg26513634	1.000	57.6	13.6	78.3
cg26717763	1.000	58.8	81.8	26.7
cg27071793	1.000	62.8	63.6	38.3
cg27084232	1.000	71.7	22.7	46.7
cg27258561	1.000	54.1	59.1	43.3
cg27342837	1.000	31.4	4.5	86.7
cg27464877	1.000	17.4	72.7	16.7
cg27506139	1.000	59.0	68.2	23.3
cg00040530	0.952	51.8	59.1	23.3
cg00234027	0.952	67.7	59.1	15.0
cg00523628	0.952	37.2	45.5	48.3
cg00539976	0.952	35.7	0.0	85.0
cg00713204	0.952	43.7	54.5	51.7
cg00904966	0.952	19.4	18.2	78.3
cg01050429	0.952	20.9	45.5	41.7
cg01496720	0.952	27.0	0.0	88.3
cg01646268	0.952	70.5	4.5	86.7
cg01856162	0.952	40.5	4.5	86.7
cg01987353	0.952	58.5	9.1	78.3
cg03315407	0.952	41.8	0.0	90.0
cg03555881	0.952	70.0	81.8	28.3
cg04048392	0.952	24.2	54.5	53.3
cg04362790	0.952	74.4	0.0	86.7
cg04473654	0.952	52.5	0.0	90.0
cg04741128	0.952	81.9	54.5	21.7
cg04938780	0.952	54.0	0.0	81.7
cg05206884	0.952	21.3	0.0	85.0
cg06452129	0.952	62.1	27.3	58.3
cg06797925	0.952	10.8	18.2	66.7
cg06837568	0.952	50.5	9.1	85.0
cg07040661	0.952	78.0	45.5	45.0
cg07093428	0.952	81.9	63.6	38.3
cg07366670	0.952	40.2	0.0	78.3
cg07676859	0.952	30.5	4.5	81.7
cg07937427	0.952	43.2	0.0	90.0
cg08021508	0.952	78.0	50.0	31.7
cg08071329	0.952	80.3	13.6	38.3
cg08449558	0.952	85.4	27.3	38.3
cg09165004	0.952	24.6	22.7	71.7
cg09919917	0.952	66.8	4.5	90.0
cg11161597	0.952	51.8	36.4	53.3
cg11389172	0.952	22.4	13.6	78.3
cg11401214	0.952	22.3	40.9	33.3
cg11583907	0.952	47.9	36.4	66.7
cg11645453	0.952	43.2	63.6	33.3
cg11805111	0.952	31.1	9.1	88.3
cg11859398	0.952	59.8	4.5	85.0
cg11950383	0.952	22.6	9.1	85.0
cg13016797	0.952	48.1	54.5	25.0
cg13380890	0.952	73.3	68.2	15.0
cg14358088	0.952	44.6	9.1	90.0
cg14361252	0.952	58.6	45.5	53.3
cg15534212	0.952	30.8	0.0	88.3
cg16047279	0.952	47.2	0.0	90.0
cg16398051	0.952	32.4	77.3	28.3
cg16541353	0.952	60.1	40.9	36.7

cg17292337	0.952	25.0	59.1	31.7
cg17657179	0.952	25.2	13.6	78.3
cg18006637	0.952	49.5	0.0	88.3
cg18052547	0.952	29.9	40.9	53.3
cg18057887	0.952	80.8	40.9	63.3
cg18093448	0.952	72.4	40.9	43.3
cg18301583	0.952	26.3	13.6	86.7
cg18343437	0.952	25.9	4.5	88.3
cg18558969	0.952	53.2	77.3	26.7
cg18773937	0.952	46.3	0.0	90.0
cg19180624	0.952	21.7	13.6	70.0
cg19806642	0.952	33.6	0.0	88.3
cg19825302	0.952	41.7	9.1	83.3
cg19980648	0.952	53.8	50.0	48.3
cg20102280	0.952	53.8	9.1	71.7
cg20246113	0.952	37.6	22.7	48.3
cg21234561	0.952	8.9	68.2	33.3
cg21350575	0.952	18.1	9.1	85.0
cg21795255	0.952	52.5	68.2	35.0
cg21918313	0.952	42.1	9.1	81.7
cg22068400	0.952	24.9	40.9	50.0
cg22301128	0.952	45.2	0.0	83.3
cg22532079	0.952	26.2	13.6	53.3
cg22670147	0.952	23.8	9.1	83.3
cg22946150	0.952	25.4	22.7	73.3
cg22955778	0.952	41.7	31.8	80.0
cg22993154	0.952	34.7	36.4	58.3
cg23602092	0.952	36.2	0.0	83.3
cg24366211	0.952	50.8	0.0	90.0
cg24416513	0.952	19.5	0.0	86.7
cg25025181	0.952	70.0	4.5	88.3
cg25197698	0.952	70.6	31.8	50.0
cg25372449	0.952	29.3	45.5	50.0
cg25477839	0.952	73.4	18.2	66.7
cg25907132	0.952	26.0	54.5	41.7
cg26035071	0.952	77.8	63.6	35.0
cg26416887	0.952	48.3	40.9	53.3
cg26454724	0.952	63.9	68.2	33.3
cg26502852	0.952	31.9	0.0	90.0
cg26764761	0.952	78.0	27.3	63.3
cg26825341	0.952	61.4	31.8	66.7
cg27046936	0.952	31.4	18.2	61.7
cg27102737	0.952	77.7	63.6	30.0
cg27205904	0.952	21.6	68.2	31.7
cg27237300	0.952	16.0	9.1	81.7
cg27359566	0.952	46.5	0.0	88.3
cg27473416	0.952	32.9	68.2	40.0
cg27628891	0.952	85.5	54.5	25.0
cg00049868	0.905	66.8	4.5	85.0
cg00288598	0.905	72.7	0.0	91.7
cg00574919	0.905	50.5	63.6	26.7
cg00690554	0.905	60.1	0.0	91.7
cg00942219	0.905	33.3	18.2	78.3
cg01291336	0.905	40.1	0.0	85.0
cg01462799	0.905	44.8	63.6	16.7
cg01966816	0.905	21.2	22.7	76.7
cg02004499	0.905	78.1	50.0	41.7
cg02119792	0.905	28.0	0.0	83.3

cg02201753	0.905	50.3	31.8	50.0
cg02378847	0.905	26.4	18.2	75.0
cg02502145	0.905	28.4	36.4	56.7
cg03195377	0.905	48.6	27.3	65.0
cg03232620	0.905	21.8	0.0	80.0
cg03263543	0.905	60.7	50.0	51.7
cg03308092	0.905	37.0	9.1	85.0
cg03450948	0.905	27.1	22.7	68.3
cg03495753	0.905	34.1	18.2	73.3
cg04986567	0.905	69.2	36.4	71.7
cg05056638	0.905	48.9	13.6	78.3
cg05099387	0.905	28.9	18.2	70.0
cg05392448	0.905	68.2	54.5	65.0
cg05649391	0.905	78.1	13.6	75.0
cg05792169	0.905	50.4	0.0	91.7
cg05925327	0.905	20.3	40.9	73.3
cg05981038	0.905	36.9	0.0	90.0
cg06183338	0.905	23.1	0.0	91.7
cg06304097	0.905	30.0	0.0	88.3
cg06612088	0.905	34.8	0.0	80.0
cg06683094	0.905	59.8	40.9	63.3
cg06693983	0.905	58.1	31.8	65.0
cg06766860	0.905	43.5	40.9	43.3
cg06946797	0.905	46.3	0.0	91.7
cg07249765	0.905	54.6	54.5	40.0
cg07365741	0.905	62.7	68.2	36.7
cg07496207	0.905	61.8	22.7	76.7
cg07565505	0.905	29.6	0.0	91.7
cg08057896	0.905	75.7	50.0	53.3
cg08124399	0.905	72.3	63.6	35.0
cg08208899	0.905	68.8	0.0	90.0
cg08315202	0.905	22.1	0.0	85.0
cg08475953	0.905	28.4	0.0	88.3
cg08750459	0.905	41.2	63.6	33.3
cg09232555	0.905	63.2	18.2	81.7
cg09557047	0.905	49.8	45.5	30.0
cg09887589	0.905	22.7	0.0	90.0
cg09916840	0.905	23.5	68.2	26.7
cg10070328	0.905	60.5	36.4	53.3
cg10317314	0.905	25.7	45.5	45.0
cg10863737	0.905	53.5	63.6	25.0
cg10926851	0.905	69.4	18.2	58.3
cg10958452	0.905	79.6	13.6	83.3
cg11116086	0.905	48.1	0.0	91.7
cg11219411	0.905	44.3	40.9	43.3
cg11821245	0.905	44.1	59.1	46.7
cg12164282	0.905	30.9	0.0	86.7
cg12453905	0.905	29.4	0.0	90.0
cg12456714	0.905	23.8	0.0	88.3
cg12591668	0.905	52.3	0.0	88.3
cg12868970	0.905	46.1	4.5	85.0
cg13125506	0.905	60.8	31.8	71.7
cg13207180	0.905	59.8	59.1	16.7
cg13327911	0.905	45.0	0.0	91.7
cg13502540	0.905	52.4	50.0	25.0
cg13699887	0.905	22.3	0.0	91.7
cg13794530	0.905	17.6	0.0	86.7
cg14040131	0.905	80.2	9.1	83.3

cg14181874	0.905	63.0	36.4	48.3
cg14687298	0.905	30.4	50.0	43.3
cg14691985	0.905	50.7	68.2	11.7
cg14766700	0.905	14.1	77.3	38.3
cg14769786	0.905	28.2	54.5	40.0
cg15181598	0.905	24.3	31.8	71.7
cg15302032	0.905	46.1	13.6	73.3
cg15954684	0.905	4.9	77.3	15.0
cg16051083	0.905	7.6	27.3	48.3
cg16268315	0.905	69.7	9.1	88.3
cg16331745	0.905	22.1	59.1	23.3
cg16663419	0.905	58.5	4.5	85.0
cg17352152	0.905	36.9	4.5	85.0
cg17373649	0.905	91.3	45.5	38.3
cg17947175	0.905	77.5	18.2	48.3
cg18197392	0.905	18.7	4.5	83.3
cg18277682	0.905	86.1	22.7	71.7
cg18384778	0.905	30.9	0.0	91.7
cg18460830	0.905	53.6	45.5	30.0
cg19747632	0.905	20.9	4.5	88.3
cg19923650	0.905	23.4	0.0	88.3
cg19974223	0.905	63.6	45.5	73.3
cg20047561	0.905	71.7	31.8	65.0
cg20680163	0.905	60.2	0.0	88.3
cg20978923	0.905	47.8	18.2	90.0
cg21838924	0.905	28.7	40.9	51.7
cg22534145	0.905	29.9	9.1	81.7
cg22710716	0.905	8.1	59.1	16.7
cg22784954	0.905	29.4	4.5	86.7
cg23224356	0.905	15.8	81.8	5.0
cg23420260	0.905	24.4	22.7	68.3
cg23434815	0.905	69.4	0.0	88.3
cg23822445	0.905	59.0	9.1	86.7
cg23872487	0.905	66.3	45.5	21.7
cg24150172	0.905	20.4	4.5	81.7
cg24221541	0.905	22.9	18.2	76.7
cg24506221	0.905	75.8	45.5	31.7
cg24530147	0.905	61.9	18.2	85.0
cg24686902	0.905	47.9	59.1	13.3
cg24809973	0.905	26.2	0.0	90.0
cg24881973	0.905	49.0	4.5	71.7
cg25139649	0.905	59.4	4.5	85.0
cg25189564	0.905	24.7	0.0	85.0
cg25284397	0.905	49.3	63.6	45.0
cg25999442	0.905	18.4	9.1	85.0
cg26122980	0.905	12.8	4.5	90.0
cg26230285	0.905	9.4	13.6	80.0
cg26376241	0.905	47.1	4.5	91.7
cg26642774	0.905	53.2	54.5	51.7
cg27081049	0.905	53.7	68.2	33.3
cg27250032	0.905	68.7	4.5	85.0
cg00754989	0.857	50.7	0.0	91.7
cg00968488	0.857	19.4	63.6	51.7
cg01105403	0.857	24.5	40.9	51.7
cg01161842	0.857	74.3	59.1	11.7
cg01296877	0.857	55.4	72.7	40.0
cg01710189	0.857	43.6	0.0	91.7
cg02091185	0.857	45.6	22.7	66.7

cg02467990	0.857	20.6	4.5	73.3
cg03221390	0.857	24.7	22.7	61.7
cg04688450	0.857	42.4	77.3	36.7
cg04875706	0.857	60.6	68.2	21.7
cg05651243	0.857	69.3	36.4	46.7
cg06797068	0.857	69.7	63.6	35.0
cg06836319	0.857	57.9	40.9	61.7
cg07150062	0.857	31.0	13.6	70.0
cg07366506	0.857	90.2	86.4	20.0
cg09768983	0.857	55.1	63.6	36.7
cg10052190	0.857	12.4	54.5	25.0
cg11018337	0.857	14.0	50.0	45.0
cg11211965	0.857	53.1	18.2	73.3
cg11706129	0.857	57.8	27.3	61.7
cg11969330	0.857	50.0	22.7	40.0
cg12045875	0.857	79.9	63.6	30.0
cg12623145	0.857	30.4	68.2	15.0
cg12711252	0.857	38.9	9.1	80.0
cg13197406	0.857	2.9	68.2	31.7
cg13312702	0.857	19.7	4.5	86.7
cg13917577	0.857	46.7	36.4	31.7
cg15447512	0.857	12.4	40.9	68.3
cg15969227	0.857	51.9	22.7	65.0
cg16060930	0.857	88.9	90.9	5.0
cg16293892	0.857	71.1	59.1	45.0
cg16464924	0.857	34.4	27.3	53.3
cg17302580	0.857	65.5	40.9	60.0
cg17763566	0.857	58.6	50.0	46.7
cg18010131	0.857	72.3	0.0	85.0
cg18117367	0.857	19.9	22.7	81.7
cg18391209	0.857	20.3	72.7	10.0
cg19174643	0.857	47.0	0.0	91.7
cg19564375	0.857	53.4	68.2	33.3
cg20704963	0.857	51.0	36.4	61.7
cg20826526	0.857	68.7	68.2	20.0
cg21243597	0.857	39.0	0.0	91.7
cg21247722	0.857	42.0	54.5	20.0
cg23200509	0.857	58.4	36.4	80.0
cg23732978	0.857	52.9	36.4	35.0
cg23786747	0.857	74.9	27.3	60.0
cg24221965	0.857	58.9	13.6	81.7
cg24415208	0.857	29.5	9.1	83.3
cg24637364	0.857	22.6	13.6	81.7
cg25181651	0.857	29.0	13.6	75.0
cg26354128	0.857	23.6	9.1	88.3
cg26398228	0.857	40.6	50.0	43.3
cg26705599	0.857	3.6	77.3	8.3
cg26873935	0.857	64.9	59.1	21.7
cg27665146	0.857	49.9	59.1	36.7
cg03590420	0.833	70.3	22.7	63.3
cg02012338	0.810	77.4	45.5	50.0
cg02188142	0.810	40.0	36.4	63.3
cg02708922	0.810	87.0	0.0	90.0
cg03216697	0.810	47.3	50.0	60.0
cg03502002	0.810	27.0	4.5	90.0
cg03848129	0.810	33.7	27.3	53.3
cg04083966	0.810	65.3	45.5	48.3
cg04888234	0.810	46.7	63.6	38.3

cg05393861	0.810	21.7	63.6	15.0
cg05740244	0.810	68.6	54.5	50.0
cg06598836	0.810	25.6	4.5	90.0
cg07025011	0.810	41.4	27.3	55.0
cg07389699	0.810	19.4	63.6	63.3
cg07791065	0.810	62.7	63.6	41.7
cg08806681	0.810	25.2	45.5	40.0
cg09907936	0.810	25.0	0.0	91.7
cg10288111	0.810	69.7	31.8	63.3
cg11680055	0.810	66.5	63.6	13.3
cg11733135	0.810	69.9	50.0	65.0
cg12697337	0.810	39.0	31.8	55.0
cg13064658	0.810	13.2	13.6	61.7
cg15360451	0.810	58.0	63.6	31.7
cg15532640	0.810	31.8	86.4	13.3
cg16255663	0.810	57.3	36.4	46.7
cg16814680	0.810	31.7	50.0	53.3
cg16827215	0.810	41.1	36.4	50.0
cg18121901	0.810	66.1	27.3	43.3
cg18464137	0.810	23.7	22.7	70.0
cg18674961	0.810	3.9	77.3	23.3
cg19816075	0.810	84.0	59.1	43.3
cg19876987	0.810	50.1	13.6	53.3
cg21202759	0.810	26.1	9.1	71.7
cg22863700	0.810	48.6	68.2	23.3
cg23349790	0.810	28.5	18.2	80.0
cg23442198	0.810	71.3	50.0	46.7
cg00084271	0.762	64.3	50.0	40.0
cg03301671	0.762	61.0	27.3	78.3
cg05344495	0.762	29.3	9.1	78.3
cg08407901	0.762	65.3	27.3	58.3
cg11097433	0.762	25.9	4.5	91.7
cg12562822	0.762	36.7	22.7	48.3
cg14645244	0.762	12.5	68.2	30.0
cg14651435	0.762	91.8	54.5	33.3
cg19695805	0.762	15.4	50.0	21.7
cg22676075	0.762	35.4	45.5	40.0
cg23513018	0.762	4.3	77.3	23.3
cg23836570	0.762	63.4	50.0	43.3
cg24153901	0.762	49.4	50.0	20.0
cg24284539	0.762	39.7	77.3	21.7
cg06378561	0.714	64.5	31.8	73.3
cg08210468	0.714	46.7	40.9	56.7
cg08354527	0.714	76.1	36.4	61.7
cg09737095	0.714	26.6	40.9	50.0
cg11612799	0.714	39.8	27.3	70.0
cg11917734	0.714	5.1	63.6	48.3
cg14218851	0.714	53.1	77.3	18.3
cg17388779	0.714	16.8	59.1	40.0
cg18189112	0.714	63.5	40.9	48.3
cg21069494	0.714	47.9	31.8	55.0
cg21810411	0.714	58.3	31.8	70.0
cg21978135	0.714	53.1	40.9	56.7
cg23404737	0.714	59.2	27.3	55.0
cg24007926	0.714	47.1	59.1	60.0
cg26773954	0.714	70.7	4.5	80.0

[a]Probe IDs for the Infinium HumanMethylation450 BeadChip (Illumina).

[b]AUC: area under the curve value obtained by receiver operating characteristic curve analysis.

[c]Based on the Youden Index.

[d]Sensitivity is defined as the ratio of the number of tissue samples diagnosed as belonging to the ER group based on the criteria relative to the exact number of patients belonging to the ER group in the initial cohort.

[e]Specificity is defined as the ratio of the number of tissue samples not diagnosed as belonging to the ER group using the criteria employed, relative to the exact number of patients belonging to the non-ER group in the initial cohort.

Supplementary Table S2 for Review. DNA methylation diagnostics for the early recurrence (ER) group based solely on Infinium data.

Probe ID[a]	DNA methylation status[b]	AUC[c]	Cutoff value (%)	Initial cohort		Validation cohort	
				Sensitivity (%)[d]	Specificity (%)[e]	Sensitivity (%)[d]	Specificity (%)[e]
cg00945409	ER < non-ER	0.814	61.0	95.5	61.7	33.3	79.2
cg07846168	ER < non-ER	0.808	56.5	81.8	76.7	23.5	73.7
cg17206555	ER < non-ER	0.807	40.9	72.7	78.3	36.4	80.0
cg02046247	ER < non-ER	0.805	75.9	90.5	66.1	50.0	87.5
cg18289710	ER < non-ER	0.798	62.6	90.9	65.0	36.8	88.2
cg09229620	ER < non-ER	0.789	35.1	95.5	56.7	36.4	92.9
cg15930703	ER < non-ER	0.785	37.7	54.6	88.3	27.8	77.8
cg21873275	ER < non-ER	0.781	85.9	95.2	56.7	23.1	70.0
cg02192855	ER > non-ER	0.781	84.1	95.5	48.3	24.0	72.7
cg19918599	ER < non-ER	0.781	65.7	90.9	55.0	42.9	86.4
cg20549290	ER < non-ER	0.777	77.8	72.7	68.3	30.0	100
cg14064694	ER > non-ER	0.776	20.1	81.8	63.3	21.7	69.2

[a]Infinium HumanMethylation450 BeadChip (Illumina) IDs of probes included in Table 2.

[b]"ER < non-ER", when the DNA methylation level of the sample was lower than the cut-off value, the sample was diagnosed as belonging to the ER group; "ER > non-ER", when the DNA methylation level of the sample was higher than the cut-off value, the sample was diagnosed as belonging to the ER group.

[c]AUC: area under the curve value obtained by receiver operating characteristic curve analysis.

[d]Sensitivity is defined as the ratio of the number of tissue samples diagnosed as belonging to the ER group based on the criteria relative to the exact number of the patients belonging to the ER group.

[e]Specificity is defined as the ratio of the number of tissue samples not diagnosed as belonging to the ER group using the criteria employed, relative to the exact number of patients belonging to the non-ER group.
