# Supplementary material for: Clinicopathological impacts of DNA methylation alterations on pancreatic ductal adenocarcinoma: prediction of early recurrence based on genome-wide DNA methylation profiling
Source: J Cancer Res Clin Oncol. 2021 Feb 26;147(5):1341–54. doi: 10.1007/s00432-021-03541-6 (PMC8021514; doi:10.1007/s00432-021-03541-6)
Supplement: Supplementary file 2 — Supplementary file2 (PDF 1462 KB) [file 432_2021_3541_MOESM2_ESM.pdf]

Table S1. Clinicopathological parameters in the initial and validation cohorts.

Clinicopathological parameters		Initial cohort (n=82)	Validation cohort (n=36)
		Mean (min-max)	
Age (years)		66.5 (27-81)	72 (45-86)
CA19-9 (U/L)		134.5 (1-14640)	72.5 (1-2392)
		Number of patients (%)	
Gender	Male	51 (62)	20 (56)
	Female	31 (38)	16 (44)
Tumor site	Head	56 (68)	19 (53)
	Body	17 (21)	10 (28)
	Tail	9 (11)	7 (19)
Differentiation	Well	8 (10)	6 (17)
	Moderate	57 (69)	24 (66)
	Poor	12 (15)	6 (17)
	Others	5 (6)	0 (0)
Tumor size (mm)	0-20	3 (4)	7 (19)
	20-40	54 (66)	23 (64)
	40-60	22 (26)	4 (11)
	60-	3 (4)	2 (6)
Lymphvascular invasion	Negative	7 (9)	3 (8)
	Positive	75 (91)	33 (92)
Perineural invasion	Negative	24 (29)	12 (33)
	Positive	58 (71)	24 (67)
Lymph node metastasis	Negative	20 (24)	7 (19)
	Positive	62 (76)	29 (81)
Surgical margin	Negative	58 (71)	20 (56)
	Microscopically positive	24 (29)	16 (44)
T factor[a]	1-2	2 (2)	1 (3)
	3	80 (98)	35 (97)
Pathological stage[a]	1A-2A	19 (23)	7 (19)
	2B	54 (66)	29 (81)
	4	9 (11)	0 (0)

[a]Based on Tumor-Node-Metastasis Classification of the International Union Against Cancer (Brierley et al, 2017).

Table S2. Primer sequences and PCR conditions for quantitative DNA methylation analysis using pyrosequencing.

Probe ID[a]	PCR primers[b]		PCR products (bp)	Sequencing primers	Target sequences [c]	Optimized PCR conditions	
	Forward	Reverse				PCR cycles	DNA polymerases
cg00945409	5'-AGTTGTTTTTTGGGGTTTTGTG-3'	5'-AAACAATAATACAACCCAACCCT -3'	140	5'-TGGTTTTGAGTTTTTAAGT-3'	5'- CG (Position 1) GTTTTATTTT CG (Position 2) GAATTTTTTAGGGTTGGGTTGTATT-3'	The following three steps are repeated 45 cycles: denaturation for 30 sec at 95 °C, annealing for 30 sec, and extension for 1 min at 72 °C. Annealing temperatures are the following: 60 °C while 5 cycles, 58 °C while 5 cycles, and then 40 cycles at 56°C.	HotStarTaq DNA polymerase (QIAGEN)
cg07846168	5'-AGAGATGAGGTTGAGAGTGAT-3'	5'-CACATTCCCCAACTTCACTTTA -3'	198	5'-ATAGTGGAGAAATAATAAG -3'	5'-TACG (Position 1) TATATTTGGTTAAGTAAATATTATT -3'	The following three steps are repeated 40 cycles: denaturation for 30 sec at 95°C, annealing for 30 sec at 52°C and extension for 1 min at 72°C.	HotStarTaq DNA polymerase (QIAGEN)
cg17206555	5'-TGTGGAGAGTTTTTGTATTAAGATG-3'	5'-TCTACCACCATTTAAATCAAAATT-3'	98	5'-GAGTGTGAGAGAGGGAA-3'	5'-ATGTTGAGT CG (Position 1) TGTGTATTATTAA CG(Position 2)-3'	The following three steps are repeated 45 cycles: denaturation for 30 sec at 95 °C, annealing for 30 sec, and extension for 1 min at 72 °C. Annealing temperatures are the following: 61 °C while 5 cycles, 58 °C while 5 cycles, and then 40 cycles at 55°C.	Amplitaq Gold 360 DNA polymerase (ThermoFisher Scientific)
cg02046247	5'-TTTTTTAGGAGTAGGAGGGAGTG-3'	5'-CTACAAAACAATCCCCAAAACCT -3'	177	5'-TATGTTTATTTGTGGAATTT -3'	5'-TT CG (Position 1) TTAGAGGGTATTAT CG(Position 2) -3'	The following three steps are repeated 45 cycles: denaturation for 30 sec at 95 °C, annealing for 30 sec, and extension for 1 min at 72 °C. Annealing temperatures are the following: 61 °C while 5 cycles, 58 °C while 5 cycles, and then 40 cycles at 55°C.	HotStarTaq DNA polymerase (QIAGEN)
cg18289710	5'-AGTGGTGATTTGTTAGGGTTAAA -3'	5'TATAAAAACCACCCCATAAACT -3'	226	5'-TGTTATTTTGAATGTTTTTG -3'	5'-TAAGGTTATTT CG (Position 1) GTAATTAAAGATATTTTAATAAAG -3'	The following three steps are repeated 45 cycles: denaturation for 30 sec at 95 °C, annealing for 30 sec, and extension for 1 min at 72 °C. Annealing temperatures are the following: 60 °C while 5 cycles, 58 °C while 5 cycles, and then 40 cycles at 56°C.	HotStarTaq DNA polymerase (QIAGEN)
cg09229620	5'-GAGAGGTGGTTTTTAGATTTGT -3'	5'-TCCCAATACAAAATCTCAACCTA -3'	239	5'-TGGGAAGAGAAGGTA -3'	5'-TTTTAAATATTTAAAT CG (Position 1) TTTTTTAT CG (Position. 2) ATTTTTGTT CG (Position 3) TTTTAATATAATGT -3'	The following three steps are repeated 40 cycles: denaturation for 30 sec at 95°C, annealing for 30 sec at 58°C and extension for 1 min at 72°C.	HotStarTaq DNA polymerase (QIAGEN)
cg15930703	5'-TAGTGGGGTTGTGAGGGTGAAGAT -3'	5'-CCAACATCTAAACACATACCACCAAC-3'	142	5'-ATATTTTATTGGTAGTTAGTAT-3'	5'-CG (Position 1)T AGTTAGGAGTTTAGGGA CG (Position 2) -3'	The following three steps are repeated 40 cycles: denaturation for 30 sec at 95°C, annealing for 30 sec at 56°C and extension for 1 min at 72°C.	HotStarTaq DNA polymerase (QIAGEN)
cg21873275	5'-TTTTTGGGTTGGTTGAGGTAG -3'	5'- AAACCCCCAAACCCATACCC-3'	138	5'-GGGGTTAGTTGGAGTTT -3'	5'-CG (Position 1) GGTGGGTATGGGTTTGG-3'	The following three steps are repeated 40 cycles: denaturation for 30 sec at 95°C, annealing for 30 sec at 56°C and extension for 1 min at 72°C.	HotStarTaq DNA polymerase (QIAGEN)
cg02192855	5'-GTTTGGGTTTATTAGTATTTTTTTGTG-3'	5'-TAATCACCCCCTTCTTAAAACCC-3'	116	5'-TTTTTTTGTGGTTATTTGA-3'	5'-CG (Position 1) GTATTATTT CG (Position 2) GTTG CG (Position 3) AATATGTTTGAATTAGTTAAGTTAG -3'	The following three steps are repeated 45 cycles: denaturation for 30 sec at 95 °C, annealing for 30 sec, and extension for 1 min at 72 °C. Annealing temperatures are the following: 60 °C while 5 cycles, 58 °C while 5 cycles, and then 40 cycles at 56°C.	HotStarTaq DNA polymerase (QIAGEN)
cg19918599	5'-TGGTAGATTTTAAGTAAGGATGTG-3'	5'-TCTACCCCAAACTACTTCTATCAA-3'	254	5'-TGTAGTGGTATAAGTGAAAG-3'	5'-GTGTAGTTTATTGAATTAG CG (Position 1) TGTTAGAAGTA CG(Position. 2) -3'	The following three steps are repeated 40 cycles: denaturation for 30 sec at 95°C, annealing for 30 sec at 54°C and extension for 1 min at 72°C.	HotStarTaq DNA polymerase (QIAGEN)
cg20549290	5'-AGATGAAAAGTGGTGTTTATTGTAAATGTAG-3'	5'-CATAACTTAAATAACAAAAACCACTTAATC-3'	338	5'-ATTTTAGTTTTTTAAGGT-3'	5'-ATTTTAGTTTAGAAT CG (Position 1) CG (Position 2) GAGATGTTTTTTTATGTTGTTTG-3'.	The following three steps are repeated 40 cycles: denaturation for 30 sec at 95°C, annealing for 30 sec at 54°C and extension for 1 min at 72°C.	Amplitaq Gold 360 DNA polymerase (ThermoFisher Scientific)
cg14064694	5'-TTGTTATTAGGGGAGTTGGTTAAA -3'	5'-TAATATACTTAATAACCCCCTTAATACCC-3'	199	5'-GGGGAGTTGGTTAAATA-3'	5'-CG (Position 1) CG (Position 2) GTGT CG (Position 3) GAGGGTATTAAGG CG (Position 4) -3'	The following three steps are repeated 45 cycles: denaturation for 30 sec at 95 °C, annealing for 30 sec, and extension for 1 min at 72 °C. Annealing temperatures are the following: 61 °C while 5 cycles, 58 °C while 5 cycles, and then 40 cycles at 55°C.	HotStarTaq DNA polymerase (QIAGEN)

[a]Probe IDs for the Infinium HumanMethylation450 BeadChip (Illumina). [b]All reverse primers were biotinylated at the 5'-end. [c]All CpG sites in target sequences are underlined and numbered sequentially from the upstream direction.

Table S3. Clinicopathological parameters of the patients belonging to epigenetic clusters.

(A) Epigenetic clustering into Clusters A, B1, B2 and B3.

Clinicopathological parameters		Cluster A (n=10)	Cluster B1 (n=8)	Cluster B2 (n=16)	Cluster B3 (n=48)	*P*-value[a]
Patients						
		Mean (min-max)				
Age (years)		64.5 (45-79)	70 (54-81)	68.5 (47-80)	65 (27-81)	0.339
CA19-9 (U/L)		47 (1-1428)	60.5 (40-2153)	369 (1-14640)	129 (1-11640)	0.491
		Number of patients (%)				
Gender	Male	5 (50)	6 (75)	11 (69)	29 (61)	0705
	Female	5 (50)	2 (25)	5 (31)	19 (40)	
Pancreatic ductal adenocarcinoma						
Tumor site	Head	8 (80)	6 (75)	10 (63)	32 (67)	0.830
	Body	2 (20)	1 (12.5)	5 (31)	9 (19)	
	Tail	0	1 (12.5)	1 (6)	7 (14)	
Differentiation	Well	1 (10)	0	3 (19)	4 (8)	0.312
	Moderate	7 (70)	7 (87.5)	10 (63)	33 (69)	
	Poor	1 (10)	0	1 (6)	10 (21)	
	Others	1 (10)	1 (12.5)	2 (12)	1 (2)	
Tumor size (mm)	0-20	0	1 (12.5)	2 (12.5)	0	0.139
	20-40	8 (80)	6 (75)	7 (43.8)	33 (69)	
	40-60	2 (20)	1 (12.5)	7 (43.8)	12 (25)	
	60-	0	0	0	3 (6)	
Lymphvascular invasion	Negative	1 (10)	3 (37.5)	1 (6)	2 (4)	<u>0.032</u>
	Positive	9 (90)	5 (62.5)	15 (94)	46 (96)	
Perineural invasion	Negative	3 (30)	3 (37.5)	4 (25)	14 (29)	0.939
	Positive	7 (70)	5 (62.5)	12 (75)	34 (71)	
Lymph node metastasis	Negative	2 (20)	5 (62.5)	4 (25)	9 (19)	0.512
	Positive	8 (80)	3 (37.5)	12 (75)	39 (81)	
Surgical margin	Negative	7 (70)	4 (50)	13 (81)	34 (71)	0.480
	Microscopically positive	3 (30)	4 (50)	3 (19)	14 (29)	
T factor[b]	1-2	0	0	3 (12.5)	0	0.082
	3	10 (100)	8 (100)	13 (87.5)	48 (100)	
Pathological stage[b]	1A-2A	2 (20)	5 (62.5)	4 (25)	9 (19)	0.246
	2B	7 (70)	3 (37.5)	9 (56)	34 (71)	
	4	1 (10)	0	3 (19)	5 (10)	

[a]Welch's t test for age and CA19-9 and Fisher's exact test for other parameters. *P* value <0.05 is underlined.
[b]Based on the Tumor-Node-Metastasis Classification of the International Union Against Cancer (Brierley et al, 2017).

(B) Epigenetic clustering into Clusters B1 and B2 and Clusters A and B3.

Clinicopathological parameters		Clusters B1 and B2 (n=24)	Clusters A and B3 (n=58)	*P*-value[a]
Patients				
		Mean (min-max)		
Age (years)		69 (47-81)	65 (27-81)	0.100
CA19-9 (U/L)		316 (1-14640)	122 (1-11640)	0.441
		Number of patients (%)		
Gender	Male	17 (71)	34 (59)	0.330
	Female	7 (29)	24 (41)	
Pancreatic ductal adenocarcinoma				
Tumor site	Head	16 (67)	40 (69)	0.805
	Body	6 (25)	11 (19)	
	Tail	2 (8)	7 (12)	
Differentiation	Well	3 (12.5)	5 (9)	0.138
	Moderate	17 (71)	40 (69)	
	Poor	1 (4)	11 (19)	
	Others	3 (12.5)	2 (2)	
Tumor size (mm)	0-20	3 (12.5)	0	<u>0.030</u>
	20-40	13 (54)	41 (71)	
	40-60	8 (33)	14 (24)	
	60-	0	3 (5)	
Lymphvascular invasion	Negative	4 (17)	3 (5)	0.186
	Positive	20 (83)	55 (95)	
Perineural invasion	Negative	7 (29)	17 (29)	1.00
	Positive	17 (71)	41 (71)	
Lymph node metastasis	Negative	9 (37)	11 (19)	0.094
	Positive	15 (63)	47 (81)	
Surgical margin	Negative	17 (71)	41 (71)	1.00
	Microscopically positive	7 (29)	17 (29)	
T factor[b]	1-2	2 (8)	0	0.083
	3	22 (92)	58 (100)	
Pathological stage[b]	1A-2A	9 (37.5)	11 (19)	0.322
	2B	12 (50)	41 (71)	
	4	3 (12.5)	6 (10)	

[a]Welch's t test for age and CA19-9 and Fisher's exact test for other parameters. *P* value <0.05 is underlined.
[b]Based on the Tumor-Node-Metastasis Classification of the International Union Against Cancer (Brierley et al, 2017).

Table S4. Clinicopathological parameters of in the early-recurrence (ER) group and non-ER group of the initial cohort.

Clinicopathological parameters		ER (n=22)	Non-ER (n=60)	*P*-value[a]
(A) Patients				
		Mean (min-max)		
Age (years)		66 (54-81)	67 (27-81)	0.912
CA19-9 (U/L)		139.5 (4-1535)	126 (1-14640)	0.660
		Number of patients (%)		
Gender	Male	13 (59)	38 (63)	0.726
	Female	9 (41)	22 (37)	
(B) Pancreatic ductal adenocarcinoma				
Tumor site	Head	15 (68)	41 (68)	0.859
	Body	4 (18)	13 (22)	
	Tail	3 (14)	6 (10)	
Differentiation	Well	0	8 (13)	0.060
	Moderate	14 (64)	43 (72)	
	Poor	6 (27)	6 (10)	
	Others	2 (9)	3 (5)	
Tumor size (mm)	0-20	0	3 (5)	0.589
	20-40	17 (77)	37 (62)	
	40-60	5 (23)	17 (28)	
	60-	0	3 (90)	
Lymphvascular invasion	Negative	1 (5)	54 (90)	0.434
	Positive	21 (95)	6 (10)	
Perineural invasion	Negative	6 (27)	42 (70)	0.810
	Positive	16 (72)	18 (30)	
Lymph node metastasis	Negative	3 (14)	17 (28)	0.170
	Positive	19 (86)	43 (72)	
Surgical margin	Negative	16 (73)	42 (70)	0.521
	Microscopically positive	6 (27)	18 (30)	
T factor[b]	1-2	0	2 (3)	0.533
	3	22 (100)	58 (97)	
Pathological stage[b]	1A-2A	3 (14)	17 (28)	0.183
	2B	14 (63)	39 (65)	
	4	5 (23)	4 (7)	

[a]Welch's t test for age and CA19-9 and Fisher's exact test for other parameters. [b]Based on the Tumor-Node-Metastasis Classification of the International Union Against Cancer (Brierley et al, 2017).

Table S5. Fifty-eight CpG sites showing area under the curve values of >0.7 in the receiver operating characteristic curves for discrimination of the early recurrence (ER) group from non-ER group based on Infinium assay.

Probe ID[a]	AUC[b]	$\|\Delta\beta_{ER\text{-}non\text{-}ER}\|$	Chromosome	Gene symbol[c]	CpG type[d]	Gene region[e]
cg00945409*	0.814	0.119	10	*ZMIZ1-AS1*	S_Shelf	Gene body
cg07846168*	0.808	0.105	13	*GPC6*	Open sea	Gene body
cg17206555*	0.807	0.107	7	*CDK14*	S_Shore	1st intron
cg02046247*	0.805	0.101	12	*NA*	Open sea	Intergenic region
cg18289710*	0.798	0.109	11	*PDGFD*	Open sea	Gene body
cg09229620*	0.789	0.106	3	*NLGN1*	S_Shore	5' UTR
cg18183817	0.787	0.110	3	*FBLN2*	Open sea	Gene body
cg21867733*	0.785	0.105	12	*NCOR2*	Open sea	5' UTR
cg15930703*	0.785	0.120	2	*DOCK10*	Open sea	Gene body
cg21873275*	0.785	0.120	1	*NBPF25P*	Open sea	Gene body
cg00498816	0.781	0.100	3	*FBLN2*	Open sea	Gene body
cg02192855*	0.781	0.110	6	*HIST1H2BI*	N_Shore	TSS200
cg19918599*	0.781	0.105	3	*NA*	Open sea	Intergenic region
cg21946824	0.781	0.106	3	*NLGN1*	S_Shore	TSS2000
cg20549290*	0.777	0.196	7	*GIMAP4*	Open sea	TSS1500
cg14064694*	0.776	0.122	6	*HIST1H2BI*	island	1st exon
cg25904344	0.773	0.107	7	*NA*	island	Intergenic region
cg08071329	0.772	0.127	6	*NA*	Open sea	Intergenic region
cg03646916	0.767	0.119	3	*ZNF167*	island	TSS200
cg22695986	0.765	0.101	7	*NA*	island	Intergenic region
cg23233200	0.764	0.105	6	*HIST1H3G*	island	TSS1500
cg23695504	0.763	0.116	1	*C1orf229*	island	1st exon
cg22969524*	0.760	0.106	17	*MPP3*	N_Shore	5' UTR
cg11744777	0.759	0.100	6	*HIST1H4K*	island	TSS200
cg02627240	0.758	0.120	7	*TPK1*	Open sea	Gene body
cg24012880	0.758	0.103	11	*TSPAN18*	Open sea	5' UTR
cg15768613	0.755	0.166	7	*ELMO1*	N_Shelf	5' UTR
cg26075208	0.752	0.101	6	*HIST1H4K*	S_Shore	TSS200
cg23764766	0.751	0.145	12	*ERC1*	Open Sea	Gene body
cg00584026	0.748	0.156	4	*TMEM175*	S_Shore	Gene body
cg11856822	0.748	0.130	1	*C1orf229*	island	1st exon
cg00660167	0.742	0.121	17	*CSNK1D*	N_Shore	Gene body
cg08554556	0.738	0.111	12	*TMEM132D*	Open sea	Gene body
cg10690440	0.736	0.142	17	*NA*	Open sea	Intergenic region
cg01517680	0.735	0.106	16	*NA*	island	Intergenic region
cg03408556	0.734	0.107	1	*C1orf229*	island	1st exon
cg00033209	0.733	0.157	16	*NAT15*	S_Shelf	5' UTR

cg05299459	0.733	0.145	2	*NA*	Open sea	Intergenic region
cg05502360	0.733	0.113	8	*KCNU1*	Open sea	TSS1500
cg08718098	0.733	0.105	12	*NA*	Open sea	Intergenic region
cg14464852	0.728	0.102	10	*TCERG1L*	Open sea	Gene body
cg16590012	0.727	0.111	1	*TAS1R3*	island	TSS1500
cg24922129	0.725	0.107	11	*NA*	N_Shore	Intergenic region
cg09324326	0.724	0.115	8	*NA*	Open sea	Intergenic region
cg11691825	0.723	0.104	1	*NA*	Open sea	Intergenic region
cg14870156	0.720	0.105	6	*HLA-DPB1*	island	Gene body
cg14498592	0.719	0.102	20	*KCNQ2*	island	Gene body
cg16991515	0.714	0.103	6	*HIST1H2BK*	N_Shore	3' UTR
cg26056277	0.714	0.110	2	*NA*	Open sea	Intergenic region
cg19566252	0.711	0.121	6	*NA*	Open sea	Intergenic region
cg04998634	0.710	0.105	19	*KLF16*	island	Gene body
cg24080129	0.705	0.153	6	*TAP2*	Open sea	Gene body
cg24961286	0.705	0.194	15	*SMAD3*	S_Shelf	Gene body
cg22777560	0.703	0.197	5	*GMCL1L*	N_Shore	Gene body
cg02772171	0.702	0.178	5	*NA*	N_Shelf	Intergenic region
cg13306870	0.702	0.107	22	*CBX7*	Open sea	3' UTR
cg00197993	0.701	0.116	6	*NA*	Open sea	Intergenic region
cg11300810	0.701	0.124	5	*NA*	Open sea	Intergenic region

[a]Probe IDs for the Infinium HumanMethylation450 BeadChip (Illumina). CpG sites included in Figure 3 and used as predictive markers are shown by asterisks.

[b]AUC: Area under the curve.

[c]NA: not annotated (located within intergenic regions).

[d]CpG islands, island shores (2000-bp regions adjacent to a CpG island) and island shelves (2000-bp regions adjacent to an island shore) are identified based on the University of California, Santa Cruz (UCSC) genome browser (https://genome.ucsc.edu/).

[e]TSS1500 (from 200 bp upstream of the transcription start site [TSS] to 1500 bp upstream of it), TSS200 (from TSS to 200 bp upstream of it), 5' UTR (untranslated region), 1st exon, 1st intron and gene body (2nd exon and downstream) are identified based on the RefSeq database (http://www.ncbi.nlm.nih.gov/refseq/).

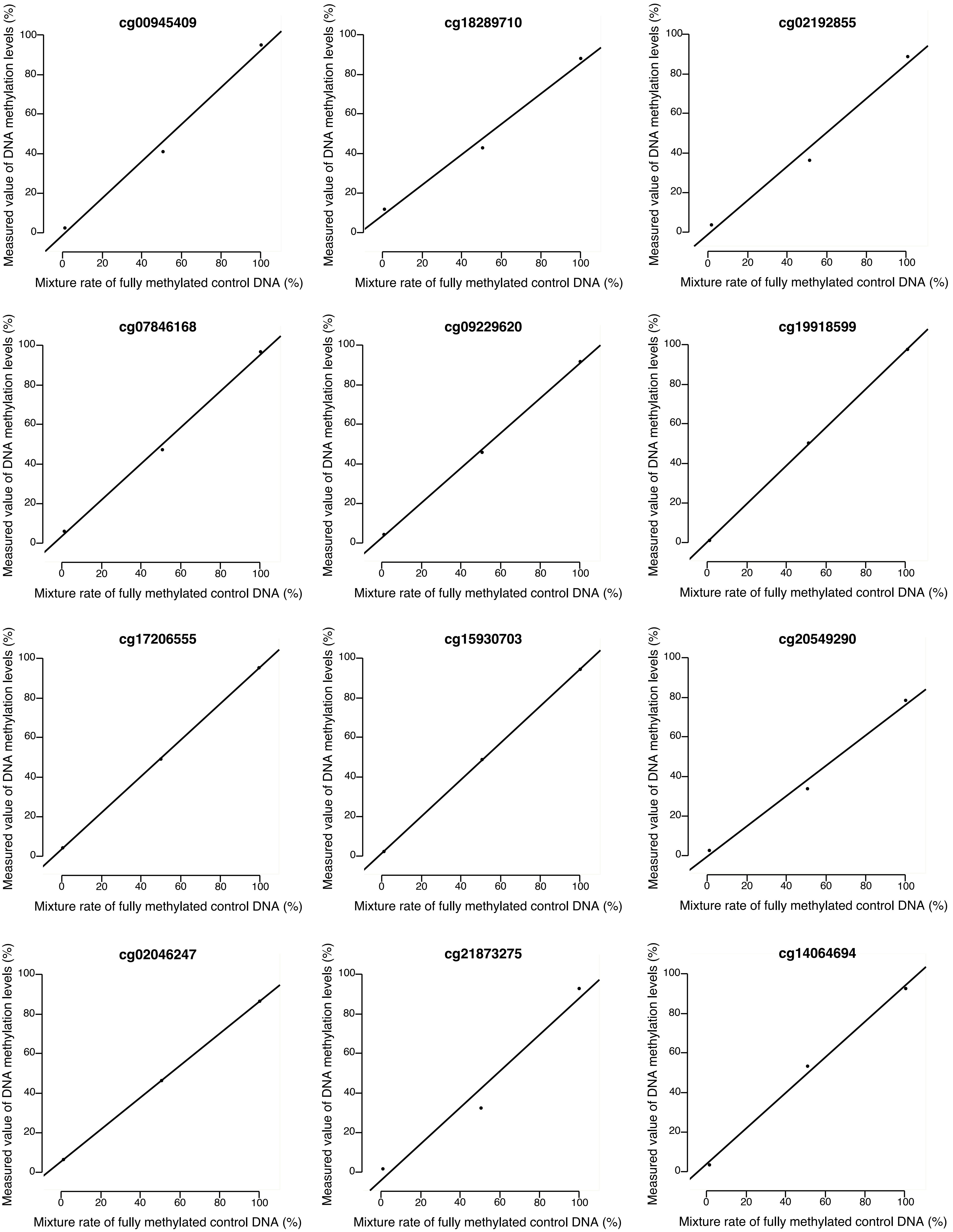

Figure S1. Optimization of PCR conditions for quantification of DNA methylation levels by pyrosequencing. The Infinium probe ID is shown at the top of each panel. 0%, 50% and 100% of fully methylated control DNA (QIAGEN) was used as a template. The linearity of the optimized protocol was successfully confirmed. The optimized PCR conditions, i.e. PCR cycle and DNA polymerase, for each primer set are summarized in Table S2.

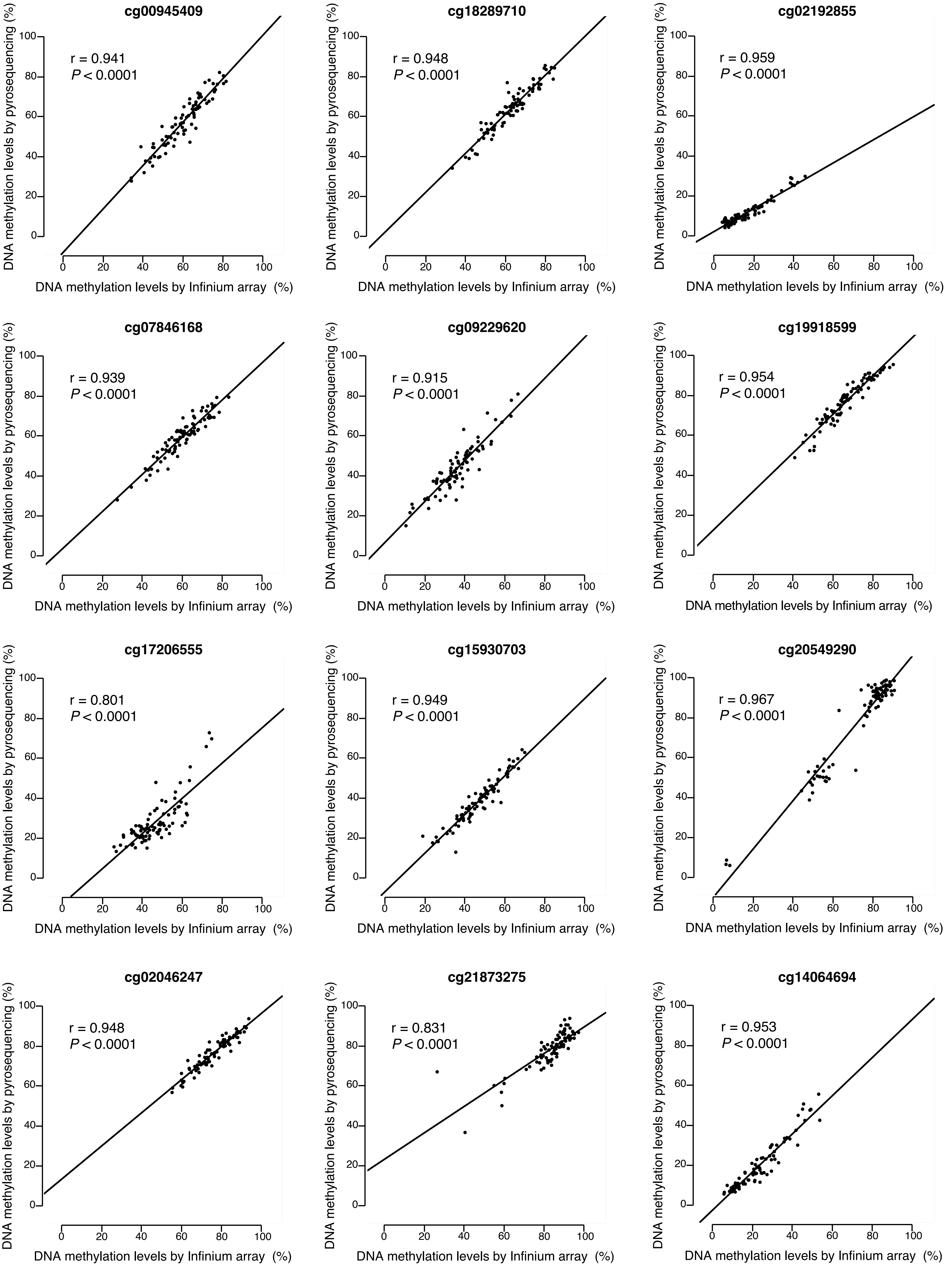

Figure S2. Correlation between DNA methylation levels based on the Infinium assay and those based on pyrosequencing using 82 cancerous tissue samples in the initial cohort. A significant correlation was successfully verified (Pearson correlation coefficients [r] ≥ 0.801 and P <0.0001).

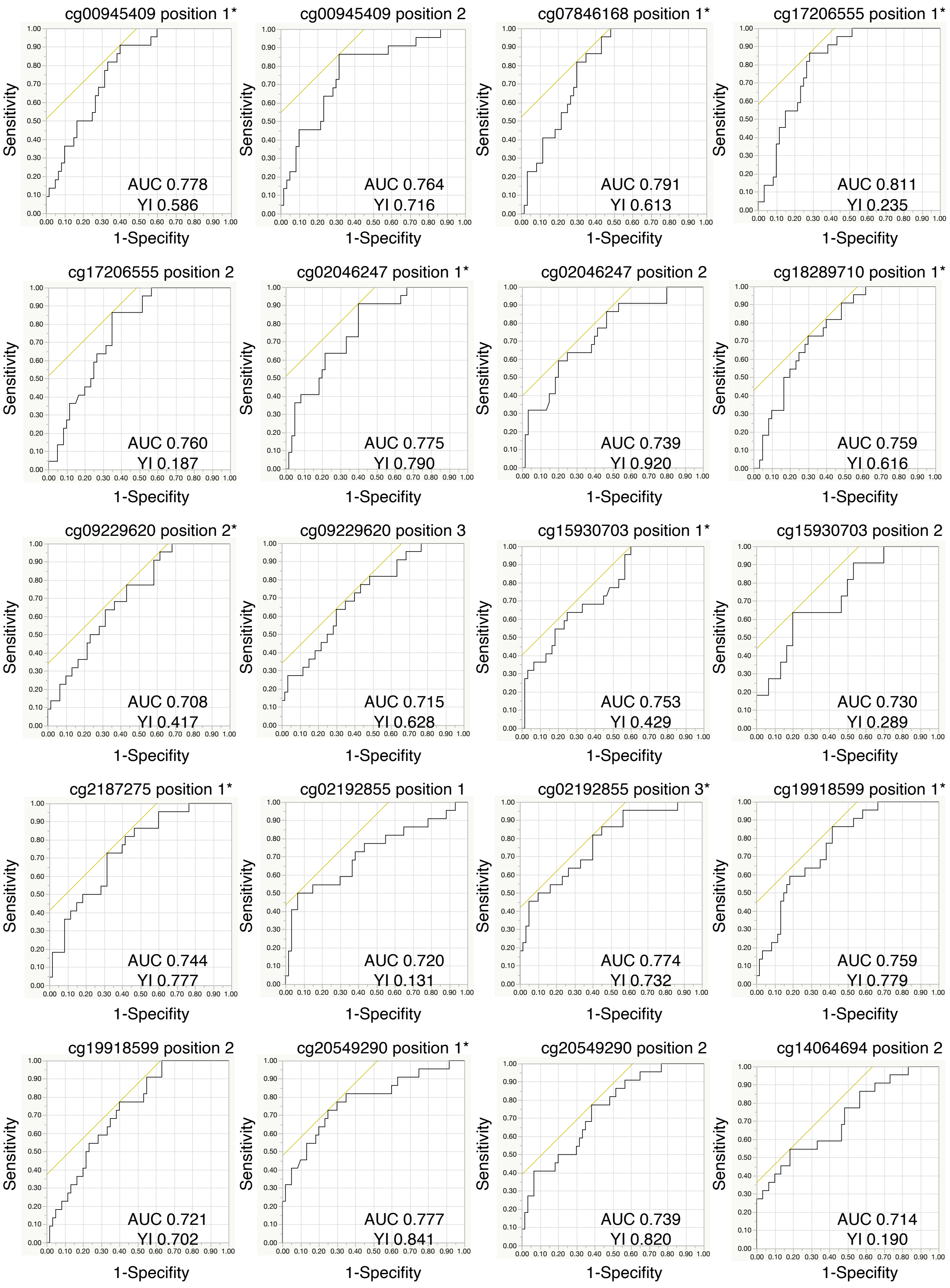

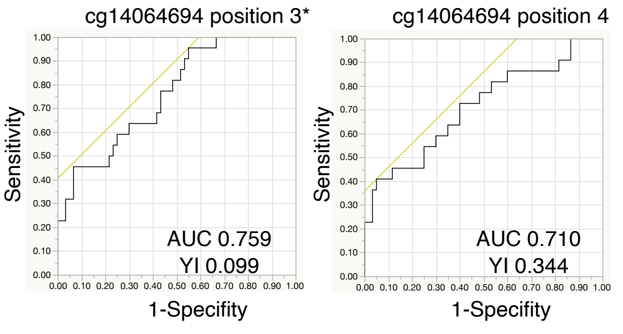

Figure S3. Receiver operating characteristic curves for discriminating the early-recurrence (ER) group (n=22) from the non-ER group (n=60) in the initial cohort using 22 candidate marker CpG sites (12 exact Infinium probe CpG sites shown with asterisks and 10 their neighboring CpG sites) based on pyrosequencing. Area under the curve (AUC) value and Youden index (YI) are shown in each panel.

(A)

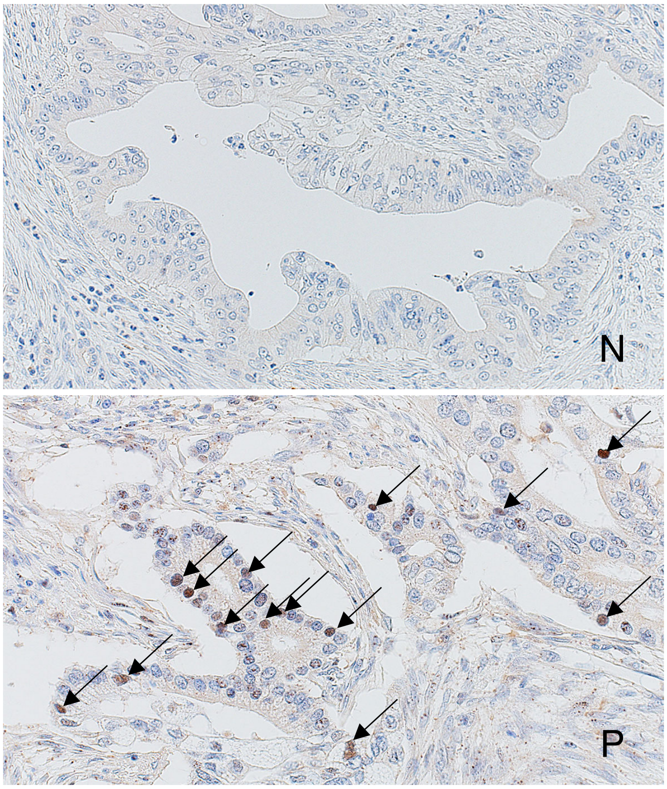

(B)

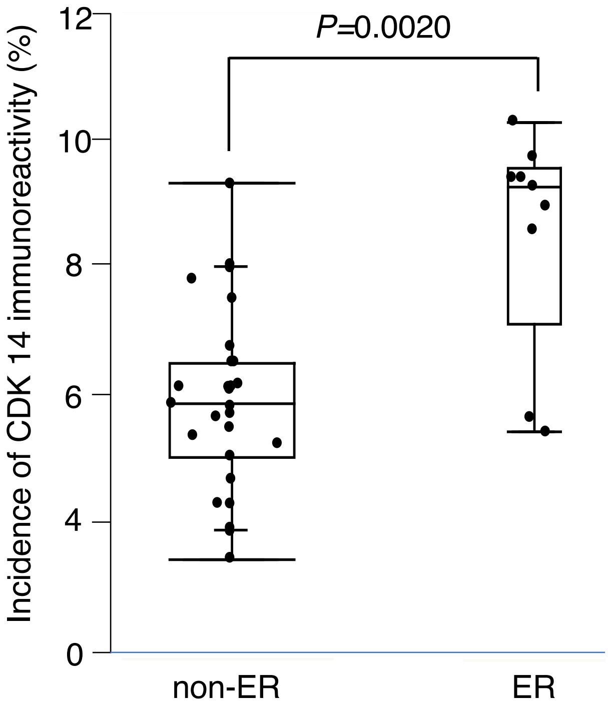

Figure S4. Immunohistochemistry for CDK14 expression in pancreatic ductal adenocarcinomas (PDACs). (A) Representative photos of a CDK14-negative area (N) and a CDK14-positive area (P). Representative nuclei of cancer cells showing immunoreactivity are indicated by arrows. (B) The incidence of nuclear immunoreactivity for CDK14 in PDACs belonging to the early recurrence (ER) group (n=9) is significantly higher than that in PDACs belonging to the non-ER group (n=27) in the validation cohort (*P*=0.0020), indicating the possibility that the level of nuclear CDK14 expression could become an ER predictor.

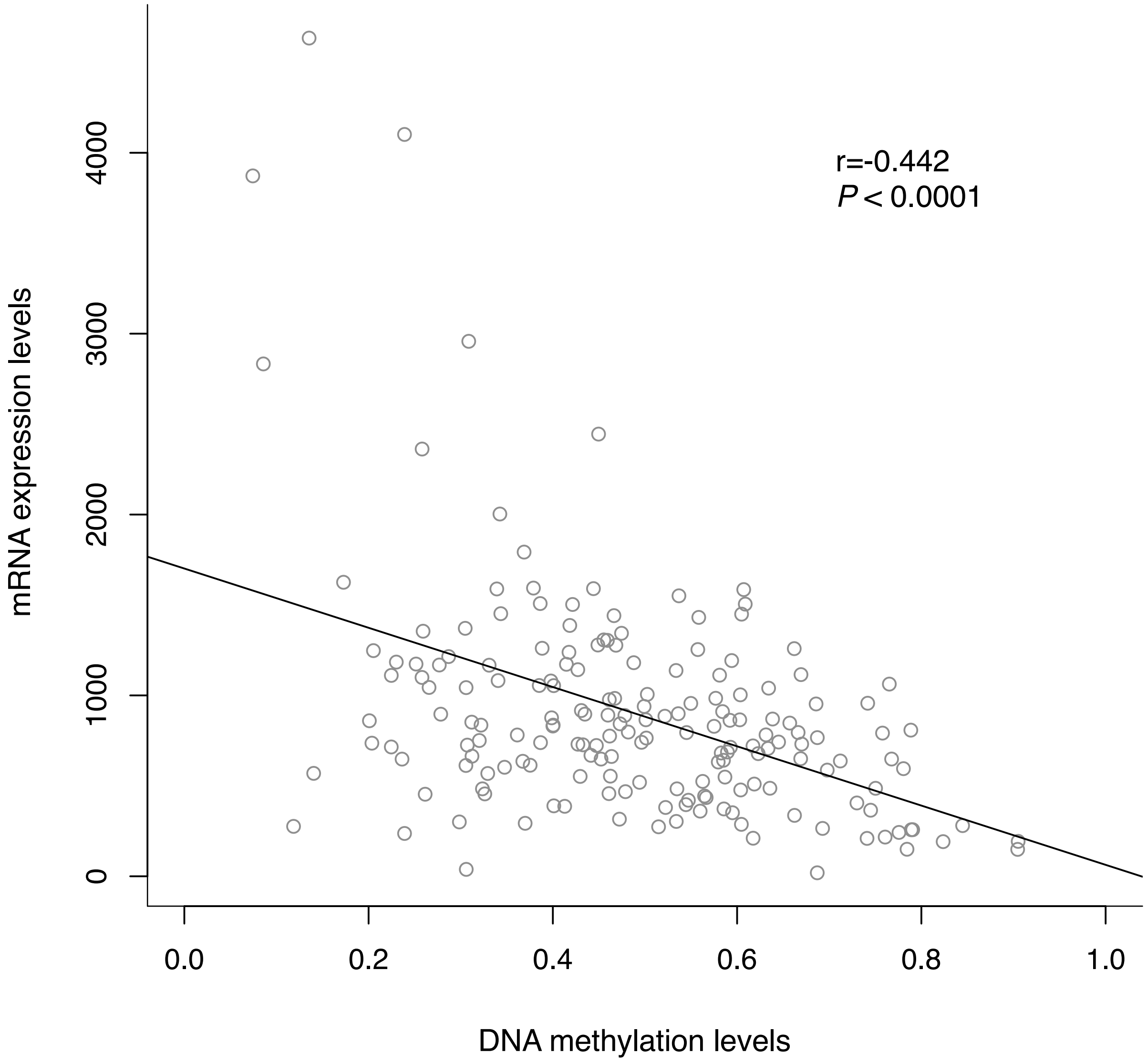

Figure S5. Significant inverse correlation between the DNA methylation level of the marker CpG site (cg17206555) and the mRNA expression level of the hypomethylated *CDK14* (cyclin-dependent kinase 14) gene based on data for cancerous and non-cancerous pancreatic tissue (n=182) deposited in the TCGA database (https://www.cancer.gov/about-nci/organization/ccg/research/structural-genomics/tcga).
